# FreeFlux: A Python Package for Time-Efficient Isotopically Nonstationary Metabolic Flux Analysis

**DOI:** 10.1021/acssynbio.3c00265

**Published:** 2023-08-10

**Authors:** Chao Wu, Michael Guarnieri, Wei Xiong

**Affiliations:** Biosciences Center, National Renewable Energy Laboratory, Golden, Colorado 80401, United States

**Keywords:** ^13^C metabolic flux analysis, isotopic labeling, steady state, transient state, labeling pattern simulation, flux estimation, python package

## Abstract

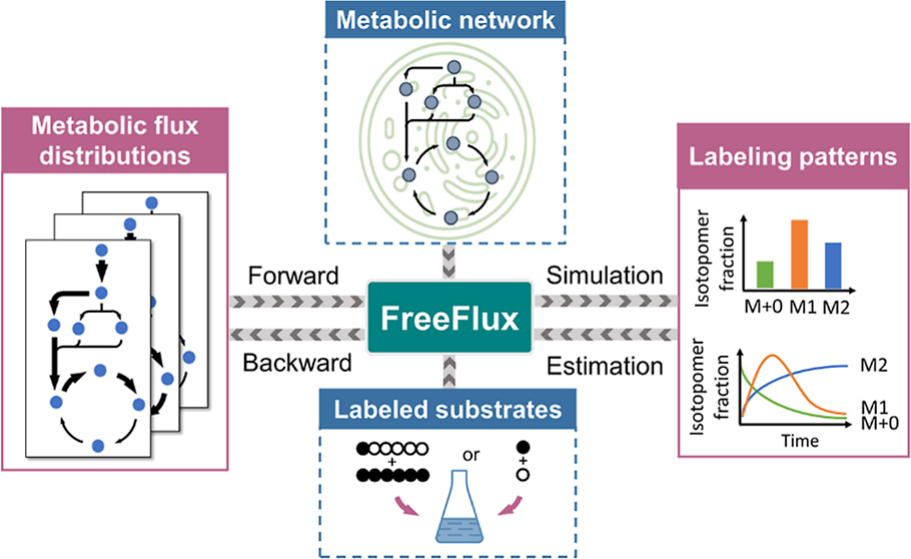

^13^C metabolic flux analysis is a powerful tool for metabolism characterization in metabolic engineering and synthetic biology. However, the widespread adoption of this tool is hindered by limited software availability and computational efficiency. Currently, the most widely accepted ^13^C-flux tools, such as INCA and 13CFLUX2, are developed in a closed-source environment. While several open-source packages or software are available, they are either computationally inefficient or only suitable for flux estimation at isotopic steady state. To address the need for a time-efficient computational tool for the more complicated flux analysis at an isotopically nonstationary state, especially for understanding the single-carbon substrate metabolism, we present FreeFlux. FreeFlux is an open-source Python package that performs labeling pattern simulation and flux analysis at both isotopic steady state and transient state, enabling a more comprehensive analysis of cellular metabolism. FreeFlux provides a set of interfaces to manipulate the objects abstracted from a labeling experiment and computational process, making it easy to integrate into other programs or pipelines. The flux estimation by FreeFlux is fast and reliable, and its validity has been confirmed by comparison with results from other computational tools using both synthetic and experimental data. FreeFlux is freely available at https://github.com/Chaowu88/freeflux with a detailed online tutorial and documentation provided at https://freeflux.readthedocs.io/en/latest/index.html.

## Introduction

^13^C metabolic flux analysis (^13^C MFA) aims to quantify the intracellular metabolic activity represented by the velocity of interconnecting biochemical reactions. It is a powerful tool used in synthetic biology, bioengineering, and medical science to understand metabolic phenotypes and the response of a natural or altered biological system.^1^ The approach involves minimizing the computed and experimentally measured labeling pattern (mass isotopomer distribution vector, MDV) of metabolites propagated via a metabolic network from isotopically labeled tracer substrates to identify the best estimate of intracellular metabolite fluxes under a quasi-steady state assumption that metabolic fluxes and isotopomer distributions are stationary.^2,3^ However, this approach is not suitable for autotrophic systems that consume single-carbon (C1) substrates such as CO_2_, CO, or CH_4_ because the labeling pattern of metabolites eventually becomes fixed irrelevant of fluxes. To solve this, isotopically nonstationary (INST) MFA was proposed, which involves measuring a series of transient MDVs at various time points to capture flux information.^4,5^ However, ^13^C INST MFA requires costly experimental and analytical procedures and presents significant computational challenges for nonspecialists, which impedes its widespread use. Several computational tools have been developed to facilitate data analysis, such as 13CFLUX2,^6^ INCA,^7^ Metran,^8^ OpenFLUX2,^9^ OpenMebius,^10^ WUflux,^11^ and FiatFlux,^12^ but they are either not open-source or built on the MATLAB platform, which can be cost-prohibitive. Additionally, most of the tools are primarily used for flux estimation at isotopic steady state. Recently, Python-based tools such as FluxPyt,^13^ mfapy,^14^ and influx_s^15^ have been developed, although increasing the computational efficiency remains a challenge for transient MFA.

As we enter the era of big data, the rate at which we are acquiring vast amounts of metabolic information is increasing rapidly. However, the development of new tools to process and analyze these data is not keeping up with this pace. Therefore, each new approach to fluxomic modeling and analysis requires corresponding advancements in tool development. We present here FreeFlux, a Python package designed to enable fast and reliable fluxomic phenotyping for both isotopically steady state and transient state. The package follows an object-oriented design, providing a collection of classes and methods for handling MDVs, metabolites, reactions, metabolic models, and conducting labeling pattern simulations and flux estimation at both isotopically steady state and nonstationary state. FreeFlux is highly flexible and can be easily integrated into other pipelines or frameworks. For instance, it can generate synthetic training data sets that relate measurable MDVs and flux distributions for machine learning or deep learning models^16^ to enable high-throughput metabolic phenotyping and can be used for validation. We assessed the performance of FreeFlux by comparing its flux estimation results at steady state and transient state with those obtained using other ^13^C MFA tools in *Escherichia coli* and *Synechocystis* models, respectively, using both synthetic and experimental data. To assist users, we provide an online tutorial and comprehensive documentation. By making this package freely available, we aim to enhance the accessibility of ^13^C fluxomics methodology to a broad user base, ultimately benefiting the community of metabolic engineering and synthetic biology at large.

## Results and Discussion

### Overview of the Python Package FreeFlux for ^13^C MFA

**Figure 1:**
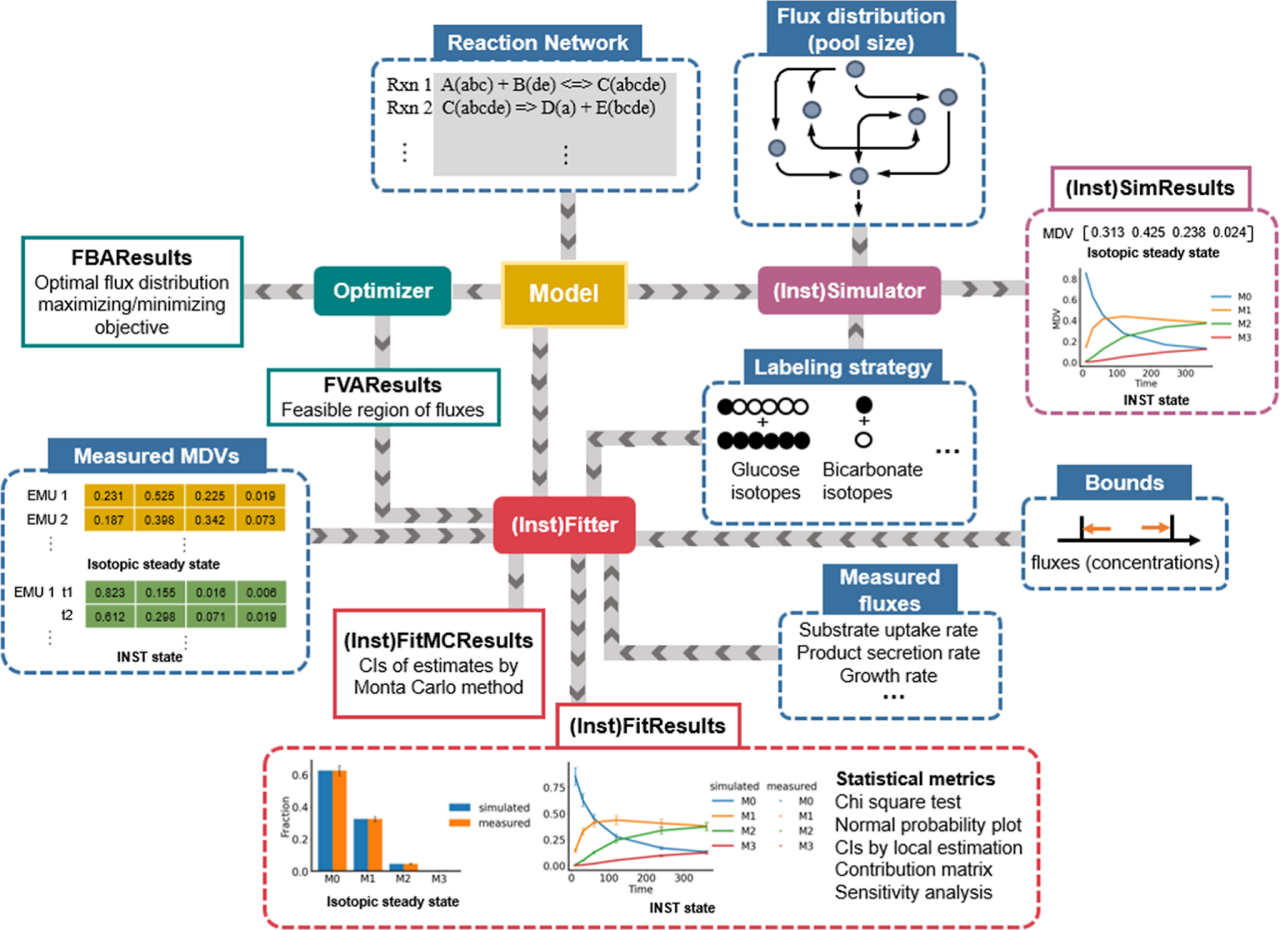
Overview of the FreeFlux dataflow. Input data are presented in blue box. Modules (classes) and their outputs are labeled with different colors. Classes in scenarios of isotopically nonstationary state have the prefix of “Inst”, as indicated in brackets. First, a network of metabolic reactions with atom mappings is provided to an instance of the Model class. The Optimizer is then called to perform FBA and FVA, with the results stored in FBAResults and FVAResults, respectively. The estimated feasible range of fluxes by FVA is used for initial guess sampling of the Fitter/InstFitter module. The Simulator module, with a defined flux distribution and labeling strategy of substrate(s), can simulate the MDV of any EMU in the network, yielding a single MDV at isotopic steady state, while the InstSimlator requires extra information of metabolite pool size and outputs a series of MDVs at ^13^C INST state. The Fitter/InstFitter module is responsible for flux estimation, which is the core function of FreeFlux. In addition to the feasible region of fluxes and defined labeling strategy, the module requires measured MDVs of metabolite fragments at single timepoint for steady state and trajectories of measured MDVs with time for ^13^C INST state, and optionally requires the measured exchange fluxes, such as substrate uptake rate, product secretion rate and/or growth rate. Specific bounds of fluxes (and concentrations) can also be set for the module. After solving a nonlinear optimization problem (see Supporting Information Methods for details), the results are stored in a FitResults/InstFitResults object, which provides methods to compare the simulated and measured MDVs and fluxes at convergence as well as methods to perform statistical tests of goodness-of-fit and local properties. The FitMCResults/InstFitMCResults object stores the uncertainties computed through the Monte Carlo method.

^13^C MFA using FreeFlux begins with building a model which consists of biochemical reactions with atom mapping indicating the correspondence of carbons in the reactants and products of each reaction (Figure 1). By calling the Optimizer object, the model is able to perform constraint-based optimizations such as flux balance analysis (FBA)^17^ and flux variability analysis (FVA).^18^ The feasible range of fluxes obtained by FVA can be used to constrain the sampling space for the initial guess in flux estimation. To simulate the MDV of metabolites, the Simulator requires a defined flux distribution and substrate labeling strategy of the metabolic network. In the case of dynamic MDVs in the ^13^C nonstationary state, additional input of metabolite pool sizes is necessary to simulate MDVs as a function of time. The core function of ^13^C MFA, flux estimation, is performed by the Fitter/InstFitter object of the model. In addition to the labeling information of the substrates, experimental measurements such as the MDVs at steady state or the time-course MDVs at transient state, as well as exchange metabolic fluxes such as the substrate uptake rate and product secretion rate, are required. If prior knowledge is available, bounds can be set for the estimates to assist in the convergence of fitting. Various statistical analyses can be conducted on the fitting results, including the χ^2^ test and normality test of the residuals. The symmetric confidence intervals (CIs) can be calculated using a local estimation method.^19^ The contribution matrix at convergence can be computed and the sensitivity analysis can be performed to evaluate the importance of measurements. Additionally, FreeFlux offers a Monte Carlo method for estimating the CIs, which provides a more accurate estimation of the uncertainties.

### Validation of Flux Estimation by FreeFlux Using Synthetic Data

**Figure 2:**
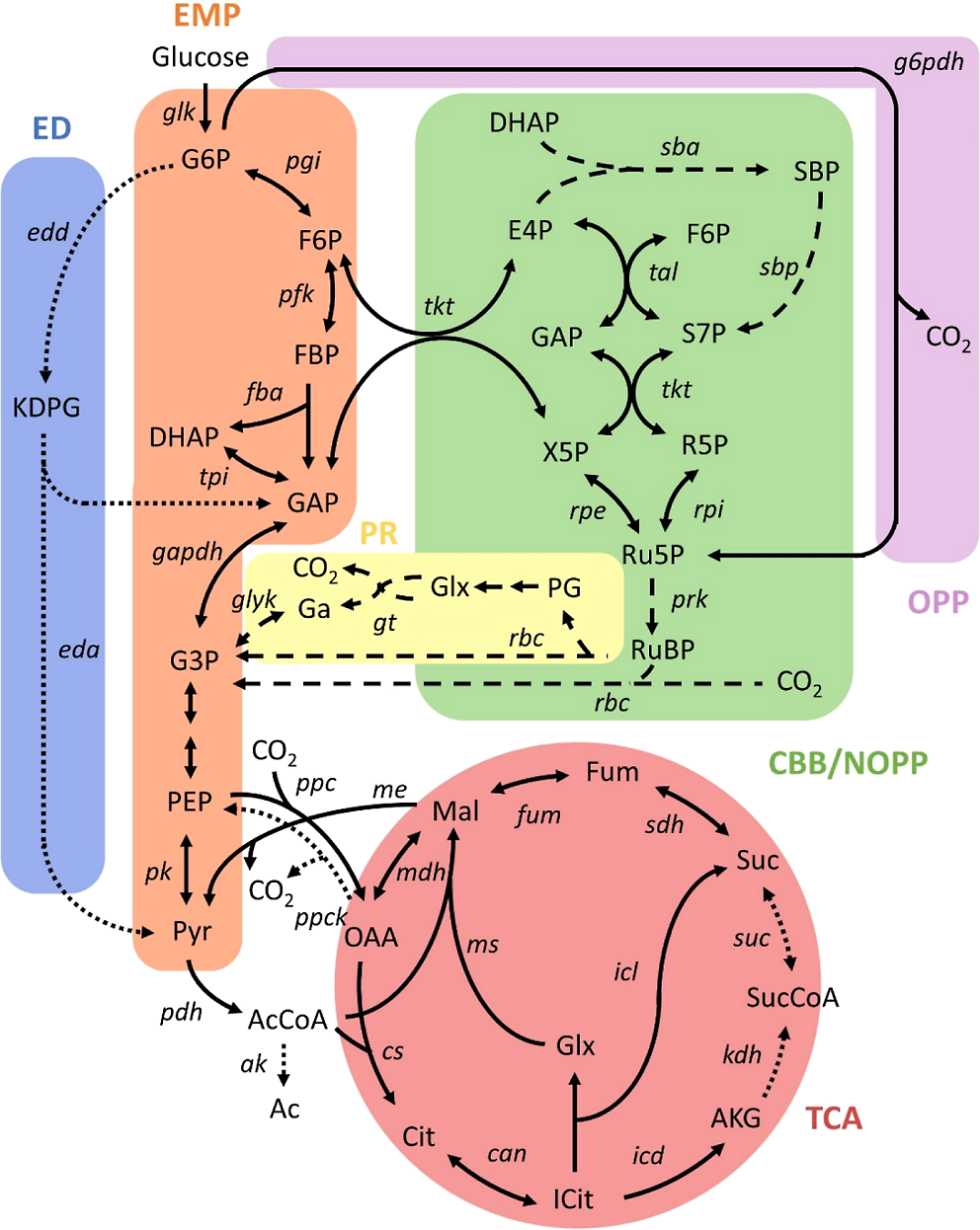
Schematic representation of central carbon metabolism in autotrophic (*Synechocystis*) and heterotrophic (*E. coli*) systems. Dotted lines represent reactions exclusive to the *E. coli* model, while dashed lines represent reactions exclusive to the *Synechocystis* model. Metabolite abbreviations: AKG, α-ketoglutarate; Ac, acetate; AcCoA, acetyl-CoA; Cit, citrate; DHAP, dihydroxyaceton phosphate; E4P, erythrose 4-phosphate; F6P, fructose 6-phosphate; FBP, fructose 1,6-bisphosphate; Fum, fumarate; G3P, 3-phosphoglycerate; G6P, glucose 6-phosphate; GAP; glyceraldehyde 3-phosphate; Ga, glycerate; Glx, glyoxylate; ICit, isocitrate; KDPG, 2-keto-3-deoxy-6-phosphogluconate; Mal, malate; OAA, oxaloacetate; PEP, phosphoenolpyruvate; PG, phosphoglycolate; Pyr, pyruvate; R5P, ribose 5-phosphate; Ru5P, ribulose 5-phosphate; RuBP, ribulose 1,5-bisphosphate; S7P, sedoheptulose 7-phosphate; SBP, sedoheptulose bisphosphate; Suc, succinate; SucCoA, succinyl-CoA; X5P, xylulose 5-phosphate. Reaction abbreviations: ak, acetate kinase; can, aconitase; cs, citrate synthase; eda, 2-keto-3-deoxy-6-phosphogluconate aldolase; edd, 6-phosphogluconate dehydratase; eno, enolase; fba, fructose-bisphosphate aldolase; fum, fumarase; g6pdh, glucose-6-phosphate dehydrogenase; gapdh, glyceraldehyde-3-phosphate dehydrogenase; glk, glucokinase; glyk, glycerate kinase; gt, glyoxylate carboligase and tartronic semialdehyde reductase; icd, isocitrate dehydrogenase; icl, isocitrate lyase; kdh, 2-oxoglutarate dehydrogenase; mdh, malate dehydrogenase; me, malic enzyme; ms, malate synthase; pdh, pyruvate dehydrogenase; pfk, phosphofructokinase; pgi, glucose-6-phosphate isomerase; pk, pyruvate kinase; ppc, phosphoenolpyruvate carboxylase; ppck, phosphoenolpyruvate carboxykinase; prk, phosphoribulokinase; rbc, ribulose-1,5-bisphosphate carboxylase/oxygenase; rpe, ribulose 5-phosphate 3-epimerase; rpi, ribose-5-phosphate isomerase; sba, sedoheptulose-1,7-bisphosphate aldolase; sbp, sedoheptulose-bisphosphatase; sdh, succinate dehydrogenase; suc, succinyl-CoA synthetase; tal, transaldolase; tkt, transketolase; tpi, triose-phosphate isomerase. Pathway abbreviations: EMP, Embden-Meyerhof-Parnas; CBB, Calvin-Benson-Bassham cycle; ED, Entner-Doudoroff; NOPP, non-oxidative pentose phosphate; OPP, oxidative pentose phosphate; PR, photorespiration; TCA.

To assess the performance of our computational tool, we first used synthetic data to conduct flux estimation using two well-established models: an *E. coli* model^20^ in isotopically steady state, and a *Synechocystis* sp. PCC6803 model^5^ in a transient state. The network reactions of both models are shown in Figure 2 and provided in Tables S1 and S6, respectively. To generate the synthetic data, we used the optGpSampler^21^ of the COBRApy toolbox^22^ to sample a reference flux distribution, and simulated the MDVs of target metabolite fragments using FreeFlux with a specified labeling strategy (we assume a constant pool size of metabolites for transient MDVs). The simulated MDVs were added with 1 mol % uncertainties. The synthetic data of *E. coli* and *Synechocystis* are listed in Tables S2, S3, S7, and S8, respectively.

**Figure 3:**
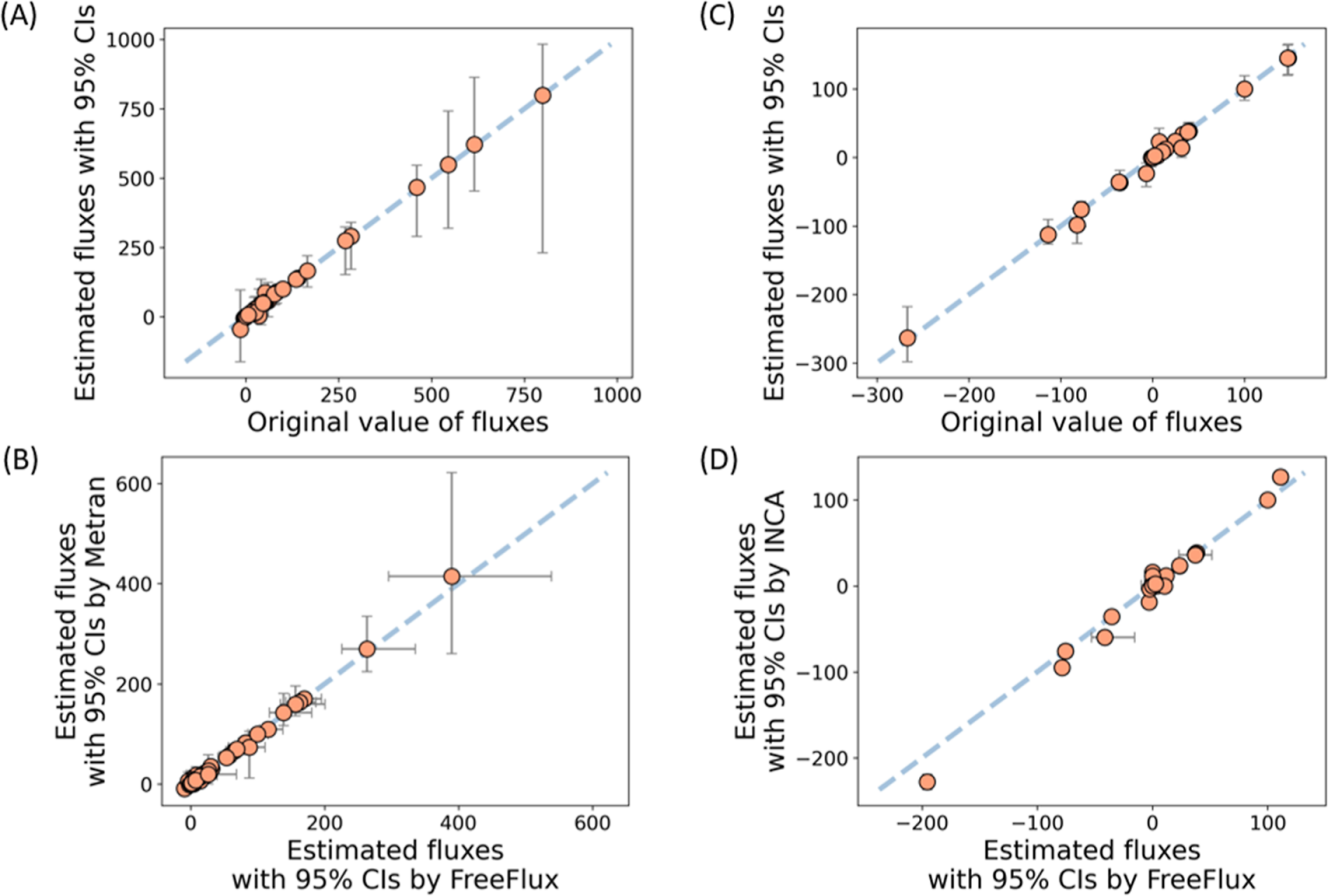
Validation of estimated fluxes by FreeFlux. (A) Comparison of estimated fluxes with the original values of synthetic data using an *E. coli* model. Synthetic data of measured MDVs and fluxes are listed in Tables S2 and S3. (B) Comparison of the estimated fluxes by FreeFlux and Metran using experimental data of *E. coli*. Experimental data of measured MDVs and fluxes are listed in Tables S4 and S5. (C) Comparison of estimated fluxes with the original values of synthetic data using a *Synechocystis* model. Synthetic data of measured MDVs and fluxes are listed in Tables S7 and S8. (D) Comparison of estimated fluxes by FreeFlux and INCA using experimental data of *Synechocystis*. Experimental data of measured MDVs and fluxes are listed in Tables S9 and S10. Fluxes of the *E. coli* model are normalized to a glucose uptake rate of 100, while fluxes of the *Synechocystis* model are normalized to a net CO2 uptake rate of 100. Error bars of FreeFlux estimates represents the 95% CIs of 500 runs by the Monte Carlo method. In flux estimation of the *Synechocystis* model using experimental data, EMU G3P_123 (fragment 459 of 3-phosphoglycerate) was not used due to data redundance. The average value was used from measurements of EMU Cit_12345 (fragment 375 and 465 of citrate) while arbitrary one of the EMU Cit_123456 from fragment 273 and 363 was chosen due to their different sampling timepoints.

As depicted in Figure 3A,C, the flux estimates for both *E. coli* and *Synechocystis* models using synthetic data show good agreement with the reference, as indicated by the small mean absolute error (MAE) of 3.0 for the *E. coli* model and 3.6 for the *Synechocystis* model, and coefficient of determination (*R*^2^) of 0.997 and 0.990, respectively. In the *E. coli* model, the three reactions with the largest estimated CIs are atpm: ATP → ATP.ex, ppc: phosphoenolpyruvate + CO_2_ → oxaloacetate and ppck: oxaloacetate + ATP → phosphoenolpyruvate + CO_2_. Stoichiometrically, ppc and ppck form a futile cycle of carbon that consumes only ATP and are coordinated with the atpm reaction to maintain the energy balance. This suggests that the estimated fluxes of these reactions are not individually constrained for a unique solution, explaining the uncertainty in their flux estimates. In the *Synechocystis* model, the CIs estimated (Figure 3C) were smaller on average compared to those in the *E. coli* model, likely due to the absence of energetic and redox-related reactions in the *Synechocystis* model. While direct comparison between the two models is not feasible due to differences in metabolites, reactions, and labeling strategies, these results suggest discrepancies in modeling can impact CI estimation.

### Validation of Flux Estimation by FreeFlux Using Experimental Data

The performance of FreeFlux was further evaluated using experimental data at isotopic steady state (*E. coli*) and transient state (*Synechocystis*) and compared with the results obtained from Metran^8^ and INCA.^7^ We obtained the experimental data for the *E. coli* model from Crown et al.,^23^ in which a mixture of [1-^13^C] glucose and [U-^13^C] glucose (4:1) was used as tracers. We corrected the experimentally measured MDVs from proteinogenic amino acids for the natural abundance of isotopomers with FreeFlux, and the corrected values are listed in Table S4. We also listed the measured fluxes in Table S5. For the *Synechocystis* model, we used the time-course MDVs experimentally measured by Gopalakrishnan et al.^24^ with natural isotope abundance corrected. The measurements used for flux estimation are provided in Tables S9 and S10. To achieve better fitting, we considered the dilution effect^25^ of intracellular CO_2_ and unlabeled amino acids for the *E. coli* model, and metabolically inactive intermediates for the *Synechocystis* model during flux estimation, consistent with previously reported data.^5,23^ The comparison of calculated and experimental MDVs at convergence of both models is provided in Figure S1 and S2, respectively.

**Figure 4:**
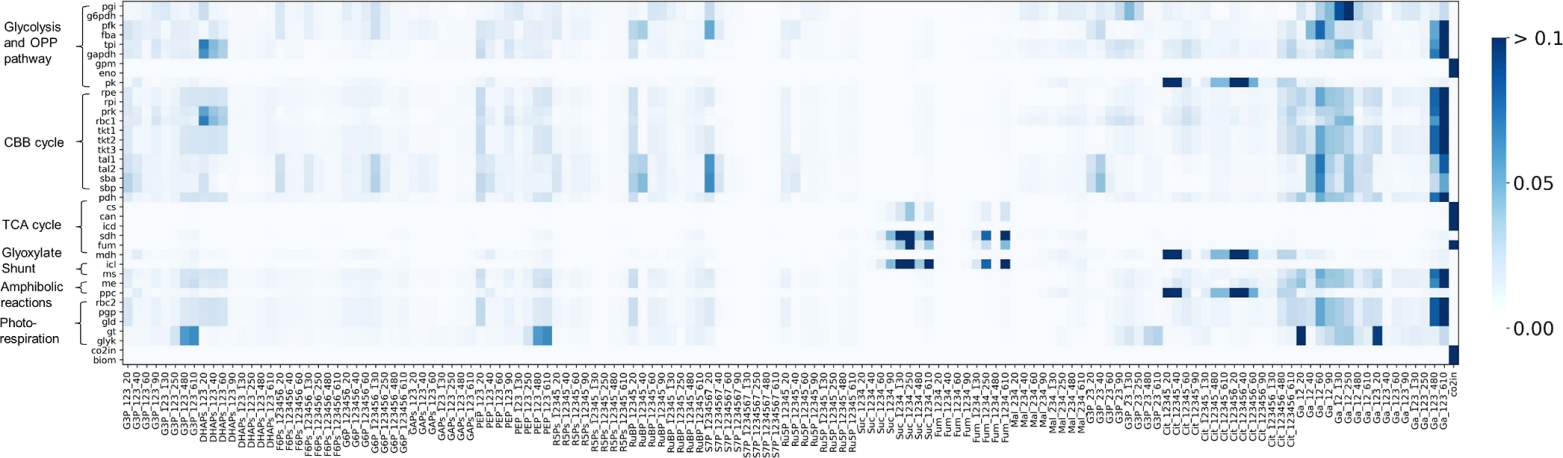
Contribution of measurement variances to the uncertainty of estimated fluxes of the *Synechocystis* model. Each cell in the heatmap represents the fractional contribution of the jth measurement to the variance of the ith flux, where *i* and *j* correspond to the row and column indices, respectively. The Measurement IDs are represented as EMU ID + timepoint. The suffix “s” after EMU ID denotes the corresponding sampling pool, as dilution effect of unlabeled pools of these metabolites were considered (Table S6). Pathway abbreviations: Please see the legend of Figure 2 for reference. Reaction abbreviations: biom, biomass formation; co2in, CO_2_ uptake; gld, glycolate dehydrogenase; gpm, phosphoglycerate mutase; pgp, phosphoglycolate phosphatase; please refer to the legend of Figure 2 for other abbreviations. Metabolite abbreviations: Please refer to the legend of Figure 2.

Figure 3B,D demonstrates a good agreement between the fluxes computed by FreeFlux and the published results obtained from other tools, with an MAE of 2.2 and 6.3 and an *R*^2^ of 0.995 and 0.974 for *E. coli* and *Synechocystis* models, respectively. These results suggest that FreeFlux is a reliable tool for flux estimation. As previously mentioned, the flux of ATP maintenance was one of the most uncertain fluxes in the *E. coli* model. Additionally, the small discrepancy in estimates between FreeFlux and INCA for the *Synechocystis* model can be attributed to the simplified inputs from the original experimental data (Figure 3D). *G*-Values estimated to account for the dilution effects in the *E. coli* model and *Synechocystis* model are provided in Tables S11 and S12, respectively, which also show good agreement with those by other tools. Based on the local sensitivities to measurements at convergence (Supporting Information Methods), we calculated a contribution matrix, which quantifies the relative impact of each measurement to the uncertainty of each flux estimate.^26^Figure 4 illustrates the significant influence that specific metabolite measurements within a pathway have on the uncertainty of reaction flux, particularly in the context of ^13^C INST MFA. For instance, the MDVs of the DHAP_123 metabolite fragment collected at the initial three timepoints (20, 40, and 60 s) account for over 10% of the variances in flux estimation for both Calvin–Benson–Bassham (CBB) cycle reactions, prk and rbc1, catalyzed by phosphoribulokinase and ribulose-1,5-bisphosphate carboxylase, as well as for the intersecting reactions, tpi and gapdh, catalyzed by triose-phosphate isomerase and glyceraldehyde-3-phosphate dehydrogenase, respectively, which connect the CBB cycle and glycolysis. Similarly, the measurement at 20 s of sedoheptulose-7-phosphate significantly impacts the CBB cycle reactions catalyzed by transaldolase (tal), sedoheptulose-1,7-bisphosphate aldolase (sba), and sedoheptulose-bisphosphatase (sbp). This evidence highlights the need for accurate determination of labeling patterns of intermediates within the initial time window in rapidly metabolizing upstream pathways. In comparison, the flux uncertainties of tricarboxylic acid (TCA) cycle reactions are primarily dictated by measurement errors of TCA metabolites, including citrate, succinate, and fumarate. Interestingly, measurements of these metabolites conducted after 90 s notably contribute to the uncertainty of pathway flux estimates, consistent with the TCA cycle’s minor metabolic activity in cyanobacteria.^27^ Moreover, photorespiration-relevant reactions (gt and glyk) that convert glyoxylate to 3-phosphoglycerate, heavily rely on the measurements of multiple metabolites including 3-phosphoglycerate, phosphoenolpyruvate and glycerate. These redundant measurements are beneficial as they reduce reliance on a single mandatory measurement of a certain metabolite (e.g., phosphoglycolate in photorespiration) for an accurate estimation of flux in the corresponding pathway. Overall, guided by the computed contribution matrix, meticulous measurements of metabolites that have a substantial impact on the pathway flux of interest become crucial for reliable analysis.

### Computation Time of Flux Estimation by FreeFlux

The computational efficiency of flux estimation using FreeFlux was evaluated using both the *E. coli* and *Synechocystis* models with synthetic and experimental data, respectively. Table 1 presents the results for single estimation and Monte Carlo CI estimation. The computation time varied significantly between synthetic and experimental data, highlighting the impact of data quality on computational efficiency. With experimental data, FreeFlux achieved flux analysis of the *Synechocystis* model in under 30 min and estimated 95% CIs in approximately 2 h (100 runs) and 8.5 h (500 runs), respectively. These time efficiencies are notable considering the typically time-intensive nature of flux estimation in the ^13^C INST state and contrast favorably against other available optimization-based tools for ^13^C MFA (Table 2). These data underscore the promise of FreeFlux as a time-efficient tool for flux estimation.

**Table 1:** Computation Time of Flux Estimation Using FreeFlux^a^

	single estimation	CI estimation (100 runs)	CI estimation (500 runs)
E. coli (synthetic data)	16 ± 2 s	2 min 22 s ± 7 s	8 min 22 s ± 11 s
E. coli (experimental data)	43 ± 7 s	3 min 48 s ± 12 s	15 min 9 s ± 24 s
Synechocystis (synthetic data)	7 min 57 s ± 11 s	1 h 1 min ± 2 min	4 h 37 min ± 6 min
Synechocystis (experimental data)	27 min 2 s ± 31 s	2 h 5 min ± 4 min	8 h 25 min ± 13 min

a Computation was performed on a high-performance computing cluster with Intel Xeon (3.0 GHz) processors using the “ralg” solver under Python 3.10 environment. CIs were estimated using Monte Carlo method of 100 and 500 runs with 30 paralleled jobs. The computation time represents the mean ± standard deviation of 10 independent trials, except for the CI estimation with 500 runs of the *Synechocystis* model, which are calculated with 5 independent trials. The *E. coli* network reactions are listed in Table S1 with synthetic and experimental data of measured MDVs and fluxes provided in Tables S2–S5, while reactions of the *Synechocystis* network are listed in Table S6 with synthetic and experimental data of measured MDVs and fluxes provided in Tables S7–S10.

**Table 2:** Optimization-Based Computational Tools Available for ^13^C MFA

name	scenario	open source	programming language	platform tested	computation time of flux estimation	refs
13CFLUX2	isotopic steady state	no	C++, Java, Python	Linux	N/A	(6)
FluxPyt	isotopic steady state	yes	Python	Windows/Linux	N/A	(13)
INCA	isotopic steady state/INST state	no	MATLAB	Windows/Linux	∼10 min single run with an E. coli model (92 reactions, 66 metabolites), ∼1 h/parameter for 95% CIs (continuation method); ∼20 min single run with a cyanobacterial model (55 reactions, 34 metabolites), ∼6 h for 95% CIs (continuation method)	(7, 32)
influx_s	isotopic steady state	yes	R, Python	Windows/Linux	3 h fixed (evolution algorithm), 15 min limited (BFGS and donlp2 algorithms) of single run with an E. coli model (68 reactions, 35 metabolites)	(15)
Metran	isotopic steady state	no	MATLAB	Windows	N/A	(8)
mfapy	isotopic steady state/INST state	yes	Python	Windows	∼0.4 h single run with an E. coli model (85 reactions); ∼1 week single run with a Synechocystis model (62 reactions)	(14)
OpenFLUX2	isotopic steady state	yes	MATLAB, Java	Windows	∼20 min single run with a Corynebacterium glutamicum model (51 reactions, 36 metabolites) (single labeling experiment), ∼30-70 h for CIs (Monte Carlo method)	(9)
OpenMebius	isotopic steady state/INST state	yes	MATLAB	Windows	7 h 42 min single run with an E. coli model (54 reactions, 22 metabolites) at INST state	(10)
WUflux	isotopic steady state	yes	MATLAB	Windows	N/A	(11)

In summary, our Python package provides a range of interfaces for accessing and manipulating the core components of MFA, granting considerable flexibility and versatility for establishing sophisticated programs or pipelines from the ground up. As demonstrated by both the *E. coli* and *Synechocystis* models, FreeFlux enables reliable and rapid flux estimation for organisms consuming single-carbon or multi-carbon substrates, making it suitable for metabolism studies of autotrophic and heterotrophic biosystems. Our package supports parallelization, which is recommended for CI calculations, particularly with large metabolic networks. FreeFlux will be continuously maintained and updated, and future versions will include new features. For instance, by incorporating heuristic methods such as evolutionary algorithms to facilitate finding the global optimum, as the current optimization solvers in FreeFlux hinge on gradient-based methods that can be sensitive to initial guesses. Furthermore, we intend to develop graphical interfaces for data input and visualization of computation results, thereby improving user-friendliness and accessibility.

## Methods and Implementation

### Model Building

FreeFlux used a model of a metabolic network consisting of biochemical reactions to perform MFA. The Model class is used to create an instance of the network, and reactions can be added individually or read from a file using the “read_from_file” method. The basic information about the reactions, such as reactant stoichiometry, reaction reversibility, and tracer atoms, should be provided. The metabolic flux of a reversible reaction is split into forward and backward flux, which are combined with the fluxes of irreversible reactions to form “total fluxes”. By specifying a distribution of total fluxes and a labeling strategy (metabolite pool sizes are also required for the transient state), the forward problem of ^13^C MFA can be solved to simulate the labeling pattern of intermediates in the network, also known as MDV. The elementary metabolite unit (EMU) method is used to establish the functional relationship between MDV and flux distribution and to identify the minimal set of precursors that contribute to the labeling pattern of a target EMU, which significantly reduces the variables and computation complexity.^28^ The EMU network decomposition is implemented using an adjacency matrix^29^ and is performed in parallel with multiple starting EMUs, which is wrapped in the “decompose_network” method. The resulting EMU networks are combined by size and stored as Pandas DataFrame objects, which can be exported as required.

### MDV Simulation

Simulation of labeling patterns is essential in optimizing the selection of isotope tracers and designing experiments for maximum information gain. The simulator requires inputs such as a defined flux distribution, metabolite concentrations, and labeling strategy. For each labeled substrate, the fraction and isotopic purity of every added isotopomer should be specified. FreeFlux supports strategies for multiple labeling substrates, for example, by the addition of a mixture of 80% [1-^13^C] glucose, 20% [U-^13^C] glucose, and 100% [U-^13^C] glutamine. The MDV simulation for the isotopic steady state is performed by the “simulate” method of a Simulator object created from the Model and an InstSimulator object for the ^13^C INST state. This yields a single isotopomer distribution and a trajectory of isotopomer distributions with time, respectively.

### Flux Estimation

Prior to flux estimation using labeling data, FreeFlux provides constraint-based analyses, that is, FBA and FVA through the Optimizer module. FBA seeks an optimal intracellular flux distribution maximizing/minimizing the objective, such as biomass formation. On the other hand, FVA is used to identify the feasible range of fluxes while maintaining an optimal or suboptimal of the objective under the same constraints of uptake and production rates as FBA. The results of FVA are stored in an FVAResults object and are used to determine the sampling region of the initial guess of flux estimation.

As the primary function of FreeFlux and the core task of ^13^C MFA, flux estimation is performed by a Fitter object. In addition to feasible flux ranges and labeling strategy, the Fitter requires inputs of measured MDV of metabolite fragments at steady state and experimental exchange fluxes such as substrate uptake rate, product secretion rate, and growth rate. Extra bounds for fluxes can also be set. By calling the “solve” method, FreeFlux attempts to find the best estimates of fluxes by minimizing the weighted residual sum of squares between simulated and measured MDVs and exchange fluxes through a nonlinear and nonconvex optimization process.^2,4,19^ Flux estimation for ^13^C INST state is accomplished by the InstFitter, which instead requires a series of MDVs measured at various time points. During the optimization process, the InstFitter solves systems of ordinary differential equations (ODEs) to simulate the transient MDVs with respect to time at each iteration (Supporting Information Methods). A noncasual first-order-hold equivalent method is used for numerical approximation of the ODE solutions which significantly improved the computation efficiency^24^ (Supporting Information Methods). FreeFlux provides two freely available solvers for the optimization problem: “ralg” and “slsqp”, which apply the r-algorithm with adaptive space dilation^30^ and the sequential least squares programming,^31^ respectively. To obtain the best flux estimation, users are encouraged to repeat calling the “solve” method with a randomized initial guess.

The fitting routine is completed when convergence criteria are satisfied, such as tolerance for termination or the maximum number of iterations. Outputs are stored in a FitResults/InstFitResults object, which provides methods for comparing the simulated and measured MDVs and fluxes. Statistical metrics, such as the χ^2^ test and normal probability plot, can be used to determine the goodness-of-fit and normality of the weighted residuals. FreeFlux calculates the estimated CIs by a local method using the Hessian matrix at convergence. The Hessian matrix is further used to determine the contribution of measurement variances to the uncertainties of estimates and the sensitivities of estimates to the perturbation of measurements.^19^ The method “solve_with_confidence_intervals” is also provided by FreeFlux to evaluate the uncertainties using Monte Carlo simulation, in which measurements are added with Gaussian noises, the computation is repeated for a specified number of times, and the CIs are obtained from quantiles of the resulting set of estimates.

As labeling experiments progress, metabolites can experience a significant reduction in ^13^C enrichment due to dilution from the inoculum, inactive intracellular metabolite pools, or media composition. To obtain a more accurate flux estimation, a dilution parameter, also known as the G-value, is incorporated to compensate for the effect.^25^ FreeFlux can calculate *G*-values for both measured metabolites, such as amino acids and intermediates, as well as unmeasured, such as CO_2_, by introducing several pseudo reactions to the network. Detailed information on how to use this feature is available in the online documentation of FreeFlux.

## MDV and Corresponding Methods

The fractional abundance of isotopomers of a metabolite fragment, also known as MDV, is a crucial computation unit for ^13^C MFA. FreeFlux provides the MDV class and a range of methods to create and manipulate an MDV instance. The “conv” method, which can also be achieved using the overloaded “*” operator, performs a convolution operation between two MDVs, yielding the merged distribution of isotopomers of substrates of a condensation reaction. Additionally, FreeFlux includes the “correct_for_inoculum” and “correct_for_natural_abundance” methods, which correct a raw measurement of MDV for the unlabeled fraction introduced by inoculum and the natural isotope abundance of specific element atoms and their numbers, respectively.^26^ The “get_natural_MDV” function calculates the natural MDV of an unlabeled EMU with n carbon atoms, while the “get_substrate_MDV” function estimates the MDV of an EMU from a labeled substrate. Notably, besides its application in ^13^C fluxomics, the MDV class can be used for data processing in qualitative labeling experiments. For more information on the functions and usage of FreeFlux, please refer to our online tutorial.

## Supporting Information Available

Implementation details of flux estimation at both isotopically steady state and nonstationary state, metabolic network with atom mapping in the *E. coli* model and the *Synechocystis* model, synthetic and experimental measurements for flux estimation in the *E. coli* model and the *Synechocystis* model, dilution parameters estimated for the *E. coli* model and the *Synechocystis* model, measured and simulated MDVs at convergence in flux estimation for the *E. coli* model and the *Synechocystis* model (PDF)

## Notes

FreeFlux is freely available at https://github.com/Chaowu88/freeflux. A detailed tutorial and documentation can be found at https://freeflux.readthedocs.io/en/latest/index.html.
