# Supplementary material for: FreeFlux: A Python Package for Time-Efficient Isotopically Nonstationary Metabolic Flux Analysis
Source: ACS Synth Biol. 2023 Aug 10;12(9):2707–14. doi: 10.1021/acssynbio.3c00265 (PMC10510750; doi:10.1021/acssynbio.3c00265)
Supplement: Supplementary file 1 — sb3c00265_si_001.pdf [file sb3c00265_si_001.pdf]

Supporting Information

FreeFlux: a Python Package for Time-Efficient Isotopically Nonstationary Metabolic Flux Analysis

Chao Wu[1]*, Michael Guarnieri[1], and Wei Xiong[1]*

1. Biosciences Center, National Renewable Energy Laboratory, Golden, Colorado 80401, United States

* Correspondence to: chao.wu@nrel.gov; wei.xiong@nrel.gov

Supplementary Methods

1 Flux estimation at isotopic steady state

1.1 The optimization problem

In ^{13}C metabolic flux analysis (^{13}C MFA) at isotopic steady state, it is essential that both metabolic flux and the labeling pattern of metabolites remain constant over time [1]. The labeling pattern is described as a mass isotopomer distribution vector (MDV) [2], which is the abundance fraction of mass isotopomers with different labeled carbon atoms. MDVs obtained from uptake of multi-carbon substrates at a single timepoint in steady state contain sufficient information to solve the fluxes in a metabolic network. Metabolite MDV can be simulated for a given labeling strategy as a function of metabolic flux, and the best estimates of fluxes are obtained by minimizing the difference between the simulated and measured MDVs. The estimated fluxes are in arbitrary units unless specific fluxes are measured, such as growth rate and exchange fluxes, which refer to the rates of substrate uptake and product secretion. The optimization problem of flux estimation in heterotrophic system can be defined as:

$$
\begin{aligned}
&\min \sum_{i=1}^{N} (\mathbf{x}_{sim,i}(\mathbf{u}) - \mathbf{x}_{exp,i})^T \boldsymbol{\Sigma}_{\mathbf{x}_{exp},i}^{-1} (\mathbf{x}_{sim,i}(\mathbf{u}) - \mathbf{x}_{exp,i}) + (\mathbf{v}_{sim}(\mathbf{u}) - \mathbf{v}_{exp})^T \boldsymbol{\Sigma}_{\mathbf{v}_{exp}}^{-1} (\mathbf{v}_{sim}(\mathbf{u}) - \mathbf{v}_{exp}) \\
&s.t.\ \ \mathbf{v} = \mathbf{N}\mathbf{u} \geq \mathbf{0} \\
&\qquad \mathbf{v}_{net}^{lb} \leq \mathbf{v}_{net} = \mathbf{T}\mathbf{v} = \mathbf{T}\mathbf{N}\mathbf{u} \leq \mathbf{v}_{net}^{ub}
\end{aligned}
\tag{1}
$$

where $\mathbf{u}$ denotes the column vector of free fluxes in length of n, and $\mathbf{N}$ is the null space of the stoichiometric matrix of the metabolic network in the shape of $m \times n$, which transforms $\mathbf{u}$ to the total fluxes vector $\mathbf{v}$ with reversible reactions split into forward and backward fluxes. The transformation matrix $\mathbf{T}$ converts $\mathbf{v}$ to net fluxes $\mathbf{v}_{net}$, which has the length of l with the range $[\mathbf{v}_{net}^{lb}, \mathbf{v}_{net}^{ub}]$. Therefore, the shape $\mathbf{T}$ is (l, m). The row vector of the ith ($i = 1, 2, ..., N$) simulated MDV, denoted by $\mathbf{x}_{sim,i}(\mathbf{u})$, represents the MDV simulated as a function of the free fluxes, and has a length of p_i. The experimentally measured MDV is presented by the vector $\mathbf{x}_{exp,i}$. The diagonal covariance matrix of measurement where all off-diagonal elements are zero, is represented by $\boldsymbol{\Sigma}_{\mathbf{x}_{exp},i}$. The simulated fluxes $\mathbf{v}_{sim}(\mathbf{u})$, measured fluxes $\mathbf{v}_{exp}$ and corresponding covariance matrix $\boldsymbol{\Sigma}_{\mathbf{v}}$ have the shape of $(1, q)$, $(1, q)$ and (q, q), respectively.

The optimization problem can be rewritten in vectorized form by combining the N MDVs into a long vector. This vectorized form is:

$$\begin{aligned} \min\ & (\mathbf{x}_{sim}(\mathbf{u})-\mathbf{x}_{exp})^T \mathbf{\Sigma}_{\mathbf{x}_{exp}}^{-1} (\mathbf{x}_{sim}(\mathbf{u})-\mathbf{x}_{exp}) + (\mathbf{v}_{sim}(\mathbf{u})-\mathbf{v}_{exp})^T \mathbf{\Sigma}_{\mathbf{v}_{exp}}^{-1} (\mathbf{v}_{sim}(\mathbf{u})-\mathbf{v}_{exp}) \\ s.t.\ & \mathbf{v} = \mathbf{N}\mathbf{u} \geq \mathbf{0} \\ & \mathbf{v}_{net}^{lb} \leq \mathbf{v}_{net} = \mathbf{T}\mathbf{v} = \mathbf{T}\mathbf{N}\mathbf{u} \leq \mathbf{v}_{net}^{ub} \end{aligned} \tag{2}$$

where the combined simulate MDVs $\mathbf{x}_{sim}(\mathbf{u})$ and measured MDVs $\mathbf{x}_{exp}$ now have a length of $\sum_i^N p_i$, and the covariance matrix of the measurement $\mathbf{\Sigma}_{\mathbf{x}_{exp}}$ has a shape of $\sum_i^N p_i \times \sum_i^N p_i$.

1.2 The gradient and the Hessian matrix

To solve the optimization problem, a sequence of quadratic programming subproblems is formulated using the first-order Taylor approximation to the simulated MDV. The solution of each subproblem indicates the search direction of the free fluxes $\mathbf{u}$ at each step [3]. To identify the direction, the Jacobian or gradient and the Hessian matrix need to be computed first by

$$gradient = (\frac{\partial \mathbf{x}_{sim}(\mathbf{u})}{\partial \mathbf{u}})^T \mathbf{\Sigma}_{\mathbf{x}_{exp}}^{-1} (\mathbf{x}_{sim}(\mathbf{u})-\mathbf{x}_{exp})^T + (\frac{\partial \mathbf{v}_{sim}(\mathbf{u})}{\partial \mathbf{u}})^T \mathbf{\Sigma}_{\mathbf{v}_{exp}}^{-1} (\mathbf{v}_{sim}(\mathbf{u})-\mathbf{v}_{exp})^T \tag{3}$$

$$hessian = (\frac{\partial \mathbf{x}_{sim}(\mathbf{u})}{\partial \mathbf{u}})^T \mathbf{\Sigma}_{\mathbf{x}_{exp}}^{-1} (\frac{\partial \mathbf{x}_{sim}(\mathbf{u})}{\partial \mathbf{u}}) + (\frac{\partial \mathbf{v}_{sim}(\mathbf{u})}{\partial \mathbf{u}})^T \mathbf{\Sigma}_{\mathbf{v}_{exp}}^{-1} (\frac{\partial \mathbf{v}_{sim}(\mathbf{u})}{\partial \mathbf{u}}) \tag{4}$$

Both gradient and Hessian consist of contributing items from MDVs and fluxes. $\frac{\partial \mathbf{x}_{sim}(\mathbf{u})}{\partial \mathbf{u}}$ is an $\sum_i^N p_i \times n$ matrix in numerator layout and $\frac{\partial \mathbf{v}_{sim}(\mathbf{u})}{\partial \mathbf{u}}$ has the shape of (q,n). The two matrices represent the sensitivity of simulation of measurable MDVs and fluxes with respect to the free fluxes.

1.3 The simulated MDVs and fluxes and their sensitivity to free fluxes

To determine the MDVs vector $\mathbf{x}_{sim}(\mathbf{u})$ in a specified flux distribution, given a metabolic network with atom mapping and labeling strategy, the Elementary Metabolite Unit (EMU) method [4] can be used to build the relation between the simulated MDVs and fluxes.

The EMUs of a metabolite consist of non-empty subsets of all its atoms (usually carbon atoms) and can be aggregated by the number of atoms. Starting from any metabolite EMU in the network, the EMU method can collect all necessary precursor labeling patterns as well as their weights (i.e., fluxes) and trace back until the substrate(s), identifying the minimal set of precursor EMUs for simulating that EMU. We have implemented the EMU network decomposition using an adjacency matrix-based method [5], and the following functional relation between fluxes and MDV of EMUs can be built using the following formular:

$$\mathbf{A}_n(\mathbf{u})\mathbf{X}_n = \mathbf{B}_n(\mathbf{u})\mathbf{Y}_n \tag{5}$$

or

$$\mathbf{AX} = \mathbf{BY} \tag{6}$$

by ignoring the independent variables $\mathbf{u}$ and size subscript n.

In this formula, $\mathbf{A}$ is a square matrix as a function of total fluxes $\mathbf{u}$ with the shape of $a \times a$, and $\mathbf{B}$ is a matrix function of $\mathbf{u}$ with the shape of $a \times b$. The MDV matrix $\mathbf{X}$ is concatenated with a MDVs with length of p, and thus has the shape of $a \times p$, whereas the $b \times p$ source MDV matrix $\mathbf{Y}$ consists of MEUs from substrates or $\mathbf{A}$ of smaller size by convolution. $\mathbf{X}$ can be estimated by

$$\mathbf{X} = \mathbf{A}^{-1}\mathbf{BY} \tag{7}$$

Taking the partial derivative with respect to $\mathbf{u}$ on both sides yields:

$$\frac{\partial \mathbf{X}}{\partial \mathbf{u}} = \mathbf{A}^{-1}(-\frac{\partial \mathbf{A}}{\partial \mathbf{u}}\mathbf{X} + \frac{\partial \mathbf{B}}{\partial \mathbf{u}}\mathbf{Y} + \mathbf{B}\frac{\partial \mathbf{Y}}{\partial \mathbf{u}}) \tag{8}$$

where $\frac{\partial \mathbf{A}}{\partial \mathbf{u}}$ has the shape of $n \times a \times a$ ($\mathbf{u}$ was assigned in the first dimension to accommodate the rule of matrix multiplication of the *matmul* function in Python package NumPy). Accordingly, $\frac{\partial \mathbf{B}}{\partial \mathbf{u}}$ has the

shape of $n \times a \times b$, and $\frac{\partial \mathbf{Y}}{\partial \mathbf{u}}$ is a $n \times b \times p$ matrix (see below for its calculation). The estimated $\frac{\partial \mathbf{X}}{\partial \mathbf{u}}$ will then have the shape of $n \times a \times p$. By combining the MDVs of $\frac{\partial \mathbf{X}}{\partial \mathbf{u}}$ at all lengths, we can obtain their sensitivity to the free fluxes ($\frac{\partial \mathbf{x}_{sim}}{\partial \mathbf{u}}$).

The calculation of $\mathbf{X}$ and $\frac{\partial \mathbf{X}}{\partial \mathbf{u}}$ starts with the smallest EMU network, in which all the MDVs in $\mathbf{Y}$ and $\frac{\partial \mathbf{Y}}{\partial \mathbf{u}}$ are derived from substrate EMUs. Without loss of generality, we assume that the matrix $\mathbf{Y}$ of some size has the form:

$$\mathbf{Y} = \begin{bmatrix} \mathbf{y}_1 \times \mathbf{y}_2 \\ \mathbf{y}_3 \\ \cdots \end{bmatrix} \tag{9}$$

Here, "×" denotes the convolution of the source MDV $\mathbf{y}_1$ and $\mathbf{y}_2$ which can be derived from either substate EMUs or EMUs of smaller size, whereas $\mathbf{y}_3$ must be a substrate MDV; otherwise, it will be moved to $\mathbf{X}$. To vectorize the calculation, the three-dimensional derivative of $\mathbf{Y}$ with respect to $\mathbf{u}$ can be defined as:

$$\frac{\partial \mathbf{Y}}{\partial \mathbf{u}} = \begin{bmatrix} \frac{\partial (\mathbf{y}_1 \times \mathbf{y}_2)}{\partial \mathbf{u}} \\ \frac{\partial \mathbf{y}_3}{\partial \mathbf{u}} \\ \cdots \end{bmatrix} = \begin{bmatrix} \frac{\partial \mathbf{y}_1}{\partial \mathbf{u}} \times \mathbf{y}_2 + \mathbf{y}_1 \times \frac{\partial \mathbf{y}_2}{\partial \mathbf{u}} \\ \frac{\partial \mathbf{y}_3}{\partial \mathbf{u}} \\ \cdots \end{bmatrix} \tag{10}$$

where $\frac{\partial \mathbf{y}_1}{\partial \mathbf{u}} \times \mathbf{y}_2$ and $\mathbf{y}_1 \times \frac{\partial \mathbf{y}_2}{\partial \mathbf{u}}$ is the 2-D convolution that takes place alongside the $\mathbf{u}$ axis, and both convolutions yield a matrix with the shape of $p \times n$. However, the substrate-originated $\mathbf{y}_3$ has zero sensitivity, i.e., $\frac{\partial \mathbf{y}_3}{\partial \mathbf{u}} = \mathbf{0}$. The derived $\frac{\partial \mathbf{Y}}{\partial \mathbf{u}}$ will have the shape of $b \times p \times n$, and will be further transformed to $n \times b \times p$ by axis swapping for multidimensional matrix multiplication.

On the other hand, the sensitivity of simulated fluxes with respect to free fluxes can be calculated by $\frac{\partial \mathbf{v}_{sim}(\mathbf{u})}{\partial \mathbf{u}} = \frac{\partial \mathbf{v}_{sim}(\mathbf{v})}{\partial \mathbf{v}} \frac{\partial \mathbf{v}}{\partial \mathbf{u}} = \frac{\partial \mathbf{v}_{sim}(\mathbf{v})}{\partial \mathbf{v}} \mathbf{N}$.

2 Flux estimation at isotopically nonstationary (INST) state

2.1 The optimization problem

The steady state ^{13}C MFA is a powerful tool for estimating metabolic fluxes in heterotrophic organisms that consume multi-carbon substrates, where the labeling patterns of intracellular metabolites at isotopic steady state can be used to infer flux distributions. However, autotrophic organisms that take up single-carbon substances like CO_2 have a fixed labeling pattern of metabolites at steady state, which makes it necessary to consider the kinetics of MDVs during the transient labeling period (isotopically nonstationary state) in order to estimate fluxes [6], In this case, the pool size of metabolites as well as the flux distribution affects the transient MDVs. The optimization problem can be formulated as follows:

$$\begin{aligned} &\min \sum_{t=1}^{M} \sum_{i=1}^{N} (\mathbf{x}_{sim,i,t}(\mathbf{p}) - \mathbf{x}_{exp,i,t})^T \mathbf{\Sigma}_{\mathbf{x}_{exp},i,t}^{-1} (\mathbf{x}_{sim,i,t}(\mathbf{p}) - \mathbf{x}_{exp,i,t}) + (\mathbf{v}_{sim}(\mathbf{p}) - \mathbf{v}_{exp})^T \mathbf{\Sigma}_{\mathbf{v}_{exp}}^{-1} (\mathbf{v}_{sim}(\mathbf{p}) - \mathbf{v}_{exp}) \\ &s.t.\ \ \mathbf{v} = \mathbf{N}\mathbf{u} \geq \mathbf{0} \\ &\qquad \mathbf{v}_{net}^{lb} \leq \mathbf{v}_{net} = \mathbf{T}\mathbf{v} = \mathbf{T}\mathbf{N}\mathbf{u} \leq \mathbf{v}_{net}^{ub} \\ &\qquad \mathbf{c} \geq \mathbf{0} \end{aligned} \tag{11}$$

The extended independent variables ($\mathbf{p}$) include both the n free fluxes ($\mathbf{u}$) and k metabolite concentrations ($\mathbf{c}$), i.e., $\mathbf{p} = \begin{bmatrix} \mathbf{u} \\ \mathbf{c} \end{bmatrix}$. To minimize the difference between the simulated and measured MDVs, the optimization problem is extended to cover the M timepoints during the transient period. A more concise and vectorized form of the problem is:

$$\begin{aligned} &\min\ (\mathbf{x}_{sim}(\mathbf{p}) - \mathbf{x}_{exp})^T \mathbf{\Sigma}_{\mathbf{x}_{exp}}^{-1} (\mathbf{x}_{sim}(\mathbf{p}) - \mathbf{x}_{exp}) + (\mathbf{v}_{sim}(\mathbf{p}) - \mathbf{v}_{exp})^T \mathbf{\Sigma}_{\mathbf{v}_{exp}}^{-1} (\mathbf{v}_{sim}(\mathbf{p}) - \mathbf{v}_{exp}) \\ &s.t.\ \ \mathbf{v} = \mathbf{N}\mathbf{u} \geq \mathbf{0} \\ &\qquad \mathbf{v}_{net}^{lb} \leq \mathbf{v}_{net} = \mathbf{T}\mathbf{v} = \mathbf{T}\mathbf{N}\mathbf{u} \leq \mathbf{v}_{net}^{ub} \\ &\qquad \mathbf{c} \geq \mathbf{0} \end{aligned} \tag{12}$$

Here, $\mathbf{x}_{sim}(\mathbf{p})$ is the concatenated row vector of simulated MDVs at all timepoints, with a length of $M\sum_{i}^{N} p_i$, and $\mathbf{x}_{exp}$ is the corresponding vector of measured MDVs. The diagonal covariance matrix $\mathbf{\Sigma}_{\mathbf{x}_{exp}}$ has the shape of $M\sum_{i}^{N} p_i \times M\sum_{i}^{N} p_i$.

2.2 The gradient and the Hessian matrix

Similar to the steady state problem, the gradient and the hessian matrix can be calculated by:

$$gradient = (\frac{\partial \mathbf{x}_{sim}(\mathbf{p})}{\partial \mathbf{p}})^T \mathbf{\Sigma}_{\mathbf{x}_{exp}}^{-1} (\mathbf{x}_{sim}(\mathbf{p}) - \mathbf{x}_{exp})^T + (\frac{\partial \mathbf{v}_{sim}(\mathbf{p})}{\partial \mathbf{p}})^T \mathbf{\Sigma}_{\mathbf{v}_{exp}}^{-1} (\mathbf{v}_{sim}(\mathbf{p}) - \mathbf{v}_{exp})^T \quad (13)$$

$$hessian = (\frac{\partial \mathbf{x}_{sim}(\mathbf{p})}{\partial \mathbf{p}})^T \mathbf{\Sigma}_{\mathbf{x}_{exp}}^{-1} (\frac{\partial \mathbf{x}_{sim}(\mathbf{p})}{\partial \mathbf{p}}) + (\frac{\partial \mathbf{v}_{sim}(\mathbf{p})}{\partial \mathbf{p}})^T \mathbf{\Sigma}_{\mathbf{v}_{exp}}^{-1} (\frac{\partial \mathbf{v}_{sim}(\mathbf{p})}{\partial \mathbf{p}}) \quad (14)$$

Here, $\frac{\partial \mathbf{x}_{sim}(\mathbf{p})}{\partial \mathbf{p}}$ and $\frac{\partial \mathbf{v}_{sim}(\mathbf{p})}{\partial \mathbf{p}}$ represent the sensitivities of simulated MDVs and fluxes with respect to both free fluxes and concentrations and have the shape of $M\sum_{i}^{N} p_i \times (n+k)$ and $q \times (n+k)$, respectively.

2.3 The simulated MDVs and fluxes and their sensitivity to free fluxes and concentrations

In the transient state, the EMU decomposition remains applicable, but the balance equations are replaced by a system of ordinary differential equations (ODEs) that describe the kinetics of labeling patterns [7]:

$$\mathbf{M}\frac{d\mathbf{X}(t)}{dt} = \mathbf{AX}(t) - \mathbf{BY}(t) \quad (15)$$

or

$$\frac{d\mathbf{X}(t)}{dt} = \mathbf{M}^{-1}\mathbf{AX}(t) - \mathbf{M}^{-1}\mathbf{BY}(t) \quad (16)$$

where $\mathbf{A}$, $\mathbf{B}$, $\mathbf{X}$ and $\mathbf{Y}$ have the same shape as in Eqn. (6), while $\mathbf{X}$ and $\mathbf{Y}$ are functions of time. The $a \times a$ diagonal matrix contains the concentrations of metabolites corresponding to the EMUs in $\mathbf{X}$. The partial derivative with respect to $\mathbf{p}$ can be computed as:

$$\frac{d}{dt}\left(\frac{\partial \mathbf{X}}{\partial \mathbf{p}}(t)\right) = \mathbf{M}^{-1}\mathbf{A}\frac{\partial \mathbf{X}}{\partial \mathbf{p}}(t) + \frac{\partial(\mathbf{M}^{-1}\mathbf{A})}{\partial \mathbf{p}}\mathbf{X}(t) - \mathbf{M}^{-1}\mathbf{B}\frac{\partial \mathbf{Y}(t)}{\partial \mathbf{p}} - \frac{\partial(\mathbf{M}^{-1}\mathbf{B})}{\partial \mathbf{p}}\mathbf{Y}(t) \quad (17)$$

Then we can derive the differential form of the sensitivity of simulated MDVs to free fluxes and concentrations. For simplification, we can define:

$$\mathbf{F} = \mathbf{M}^{-1}\mathbf{A} \quad (18)$$

$$\mathbf{G}(t) = \mathbf{M}^{-1}\mathbf{B}\mathbf{Y}(t) \quad (19)$$

$$\mathbf{H}(t) = \frac{\partial(\mathbf{M}^{-1}\mathbf{A})}{\partial \mathbf{p}}\mathbf{X}(t) - \mathbf{M}^{-1}\mathbf{B}\frac{\partial \mathbf{Y}}{\partial \mathbf{p}}(t) - \frac{\partial(\mathbf{M}^{-1}\mathbf{B})}{\partial \mathbf{p}}\mathbf{Y}(t) \quad (20)$$

Then, Eqn. (16) and (17) can be rewritten as:

$$\frac{d\mathbf{X}(t)}{dt} = \mathbf{F}\mathbf{X}(t) - \mathbf{G}(t) \quad (21)$$

$$\frac{d}{dt}\left(\frac{\partial \mathbf{X}}{\partial \mathbf{p}}(t)\right) = \mathbf{F}\frac{\partial \mathbf{X}}{\partial \mathbf{p}}(t) + \mathbf{H}(t) \quad (22)$$

This ODE systems can be solved using the integrating factor method as they have the first-order linear form [7]. After a time interval $[t, t+\Delta t]$, the simulated MDVs and their sensitivities can then be expressed as:

$$\mathbf{X}(t+\Delta t) = e^{\mathbf{F}\Delta t}\mathbf{X}(t) - \int_0^{\Delta t} e^{\mathbf{F}(\Delta t - \tau)}\mathbf{G}(t+\tau)d\tau \quad (23)$$

$$\frac{\partial \mathbf{X}}{\partial \mathbf{p}}(t+\Delta t) = e^{\mathbf{F}\Delta t}\frac{\partial \mathbf{X}}{\partial \mathbf{p}}(t) + \int_0^{\Delta t} e^{\mathbf{F}(\Delta t - \tau)}\mathbf{H}(t+\tau)d\tau \quad (24)$$

As analytical solutions are not available for the second parts of the above expressions because the convolution is discretized and nonlinear in EMU networks of different sizes, a non-causal first-order-hold equivalent

$$\mathbf{G}(t+\tau)=\mathbf{G}(t)+\frac{\tau}{\Delta t}(\mathbf{G}(t+\Delta t)-\mathbf{G}(t)) \quad (25)$$

$$\mathbf{H}(t+\tau)=\mathbf{H}(t)+\frac{\tau}{\Delta t}(\mathbf{H}(t+\Delta t)-\mathbf{H}(t)) \quad (26)$$

can be used to replace $\mathbf{G}(t+\tau)$ and $\mathbf{H}(t+\tau)$ in Eqn. (23) and (24) [8]. By integrating by parts, an approximation of the simulated MDVs and their sensitivities at $t+\Delta t$ can be obtained as follows:

$$\mathbf{X}(t+\Delta t)=\mathbf{\Phi}\mathbf{X}(t)-\mathbf{\Gamma}\mathbf{G}(t)-\mathbf{\Omega}(\mathbf{G}(t+\Delta t)-\mathbf{G}(t)) \quad (27)$$

$$\frac{\partial \mathbf{X}}{\partial \mathbf{p}}(t+\Delta t)=\mathbf{\Phi}\frac{\partial \mathbf{X}}{\partial \mathbf{p}}(t)+\mathbf{\Gamma}\mathbf{H}(t)+\mathbf{\Omega}(\mathbf{H}(t+\Delta t)-\mathbf{H}(t)) \quad (28)$$

Here, we have:

$$\mathbf{\Phi}=e^{\mathbf{F}\Delta t} \quad (29)$$

$$\mathbf{\Gamma}=(\mathbf{\Phi}-\mathbf{I})\mathbf{F}^{-1}=(e^{\mathbf{F}\Delta t}-\mathbf{I})\mathbf{F}^{-1} \quad (30)$$

$$\mathbf{\Omega}=[(\frac{\mathbf{\Gamma}}{\Delta t})-\mathbf{I}]\mathbf{F}^{-1}=[(e^{\mathbf{F}\Delta t}-\mathbf{I})(\mathbf{F}\Delta t)^{-1}-\mathbf{I}]\mathbf{F}^{-1} \quad (31)$$

The matrix exponential can be estimated using the *expm* function available in the Python package SciPy, which implements a Pade approximation method [9].

Calculation $\mathbf{X}$ of $\frac{\partial \mathbf{X}}{\partial \mathbf{p}}$ and in the transient state begins with the first sampling timepoint after the labeling experiment. Eqns. (27) and (28) are then iteratively solved to estimate the simulated MDVs and their corresponding sensitivities at various timepoints using the results of the previous timepoint as the start of a new time interval. Specifically, at the time interval $[t, t+\Delta t]$, $\mathbf{G}(t)$ and $\mathbf{H}(t)$ can be calculated using the available $\mathbf{Y}(t)$ and $\frac{\partial \mathbf{Y}}{\partial \mathbf{p}}(t)$, while computation of $\mathbf{G}(t+\Delta t)$ and $\mathbf{H}(t+\Delta t)$ depends on $\mathbf{Y}(t+\Delta t)$ and $\frac{\partial \mathbf{Y}}{\partial \mathbf{p}}(t+\Delta t)$ estimated starting from EMU network of the smallest size. Similar to the steady state estimation of $\mathbf{Y}$ and $\frac{\partial \mathbf{Y}}{\partial \mathbf{v}}$, $\mathbf{Y}(t+\Delta t)$ and $\frac{\partial \mathbf{Y}}{\partial \mathbf{p}}(t+\Delta t)$ are derived from convolution of MDVs and 2-D convolution of MDVs with their sensitivities from either substate EMUs or EMUs of smaller size at the

same timepoint. Subsequently, $\mathbf{X}(t+\Delta t)$ and $\frac{\partial \mathbf{X}}{\partial \mathbf{p}}(t+\Delta t)$ can be estimated using Eqn. (27) and (28). At the initial timepoint t_0, the matrix $\mathbf{X}(t_0)$ consists of MDV vectors representing natural abundance of isotopes, while MDVs in $\mathbf{Y}(t_0)$ represent either natural EMUs or EMUs from labeled substrate(s). $\frac{\partial \mathbf{X}}{\partial \mathbf{p}}(t_0)$ and $\frac{\partial \mathbf{Y}}{\partial \mathbf{p}}(t_0)$, however, are all zero matrices.

The sensitivity of simulated fluxes to the total independent variables ($\frac{\partial \mathbf{v}_{sim}(\mathbf{p})}{\partial \mathbf{p}}$) can be separated into two parts: sensitivity to free fluxes ($\frac{\partial \mathbf{v}_{sim}(\mathbf{p})}{\partial \mathbf{u}}$) and sensitivity to concentrations ($\frac{\partial \mathbf{v}_{sim}(\mathbf{p})}{\partial \mathbf{c}}$). Estimating $\frac{\partial \mathbf{v}_{sim}(\mathbf{p})}{\partial \mathbf{u}}$ is similar to that used in steady state MFA, resulting a $q \times n$ matrix. In contrast, since metabolic fluxes are assumed to have constant values throughout the transient state and be independent of metabolite concentrations, the $q \times k$ matrix $\frac{\partial \mathbf{v}_{sim}(\mathbf{p})}{\partial \mathbf{c}}$ is a zero matrix.

References

(1) Zamboni, N.; Fendt, S. M.; Ruhl, M.; Sauer, U. (13)C-based metabolic flux analysis. *Nat Protoc* **2009**, *4* (6), 878-892. DOI: 10.1038/nprot.2009.58.
(2) Nanchen, A.; Fuhrer, T.; Sauer, U. Determination of metabolic flux ratios from 13C-experiments and gas chromatography-mass spectrometry data: protocol and principles. *Methods Mol Biol* **2007**, *358*, 177-197. DOI: 10.1007/978-1-59745-244-1_11.
(3) Antoniewicz, M. R.; Kelleher, J. K.; Stephanopoulos, G. Determination of confidence intervals of metabolic fluxes estimated from stable isotope measurements. *Metab Eng* **2006**, *8* (4), 324-337. DOI: 10.1016/j.ymben.2006.01.004.
(4) Antoniewicz, M. R.; Kelleher, J. K.; Stephanopoulos, G. Elementary metabolite units (EMU): a novel framework for modeling isotopic distributions. *Metab Eng* **2007**, *9* (1), 68-86. DOI: 10.1016/j.ymben.2006.09.001.
(5) Wu, C.; Chen, C. H.; Lo, J.; Michener, W.; Maness, P.; Xiong, W. EMUlator: An Elementary Metabolite Unit (EMU) Based Isotope Simulator Enabled by Adjacency Matrix. *Front Microbiol* **2019**, *10*, 922. DOI: 10.3389/fmicb.2019.00922.
(6) Young, J. D.; Shastri, A. A.; Stephanopoulos, G.; Morgan, J. A. Mapping photoautotrophic metabolism with isotopically nonstationary (13)C flux analysis. *Metab Eng* **2011**, *13* (6), 656-665. DOI: 10.1016/j.ymben.2011.08.002.

(7) Young, J. D.; Walther, J. L.; Antoniewicz, M. R.; Yoo, H.; Stephanopoulos, G. An elementary metabolite unit (EMU) based method of isotopically nonstationary flux analysis. *Biotechnol Bioeng* **2008**, *99* (3), 686-699. DOI: 10.1002/bit.21632.
(8) Gopalakrishnan, S.; Pakrasi, H. B.; Maranas, C. D. Elucidation of photoautotrophic carbon flux topology in Synechocystis PCC 6803 using genome-scale carbon mapping models. *Metab Eng* **2018**, *47*, 190-199. DOI: 10.1016/j.ymben.2018.03.008. Franklin, G. F.; Powell, J. D.; Workman, M. L. *Digital control of dynamic systems*; Addison-Wesley, 1998.
(9) Al-Mohy, A. H.; Higham, N. J. A new scaling and squaring algorithm for the matrix exponential. *SIAM Journal on Matrix Analysis and Applications* **2009**, *31* (3), 970-989, Article. DOI: 10.1137/09074721X.

Supplementary Tables and Figures

Table S1. Metabolic reactions with atom mapping in the *E. coli* model.

Reaction ID	Substrate IDs(atoms)	Product IDs(atoms)	Reversibility
#Glycolysis			
pgi	G6P(abcdef)	F6P(abcdef)	1
pfk	F6P(abcdef)+ATP	FBP(abcdef)	0
fba	FBP(abcdef)	DHAP(cba)+GAP(def)	1
tpi	DHAP(abc)	GAP(abc)	1
gapdh	GAP(abc)	G3P(abc)+ATP+NADH	1
eno	G3P(abc)	PEP(abc)	1
pk	PEP(abc)	Pyr(abc)+ATP	0
#Pentose phosphate pathway			
g6pdh	G6P(abcdef)	PGluc(abcdef)+NADPH	0
gnd	PGluc(abcdef)	Ru5P(bcdef)+CO2(a)+NADPH	0
rpe	Ru5P(abcde)	X5P(abcde)	1
rpi	Ru5P(abcde)	R5P(abcde)	1
tkt1	X5P(abcde)	TK(ab)+GAP(cde)	1
tkt2	F6P(abcdef)	TK(ab)+E4P(cdef)	1
tkt3	S7P(abcdefg)	TK(ab)+R5P(cdefg)	1
tal1	F6P(abcdef)	TA(abc)+GAP(def)	1
tal2	S7P(abcdefg)	TA(abc)+E4P(defg)	1
#Entner-Doudoroff pathway			
edd	PGluc(abcdef)	KDPG(abcdef)	0
eda	KDPG(abcdef)	Pyr(abc)+GAP(def)	0
#TCA cycle			
pdh	Pyr(abc)	AcCoA(bc)+CO2(a)+NADH	0
cs	OAA(abcd)+AcCoA(ef)	Cit(efbcda)	0
can	Cit(abcdef)	ICit(abcdef)	1
icd	ICit(abcdef)	AKG(abcde)+CO2(f)+NADPH	1
kdh	AKG(abcde)	SucCoA(bcde)+CO2(a)+NADH	0
suc	SucCoA(abcd)	Suc(abcd,dcba)+ATP	1
sdh	Suc(abcd,dcba)	Fum(abcd,dcba)+FADH	1
fum	Fum(abcd,dcba)	Mal(abcd)	1
mdh	Mal(abcd)	OAA(abcd)+NADH	1
#Glyoxylate Shunt			
icl	ICit(abcdef)	Glx(ab)+Suc(edcf,fcde)	0
ms	Glx(ab)+AcCoA(cd)	Mal(abdc)	0
#Amphibolic reactions			
me1	Mal(abcd)	Pyr(abc)+CO2(d)+NADPH	0
me2	Mal(abcd)	Pyr(abc)+CO2(d)+NADH	0

ppc	PEP(abc)+CO2(d)	OAA(abcd)	0
ppck	OAA(abcd)+ATP	PEP(abc)+CO2(d)	0
#Acetic acid formation			
ak	AcCoA(ab)	Ac(ab)+ATP	0
#Amino acid biosynthesis			
gdh	AKG(abcde)+NADPH+NH4	Glu(abcde)	0
gs	Glu(abcde)+ATP+NH4	Gln(abcde)	0
pro	Glu(abcde)+ATP+2NADPH	Pro(abcde)	0
arg	Glu(abcde)+CO2(f)+Gln(ghijk)+ Asp(lmno)+AcCoA(pq)+5ATP+ NADPH	Arg(abcdef)+AKG(ghijk)+ Fum(lmno)+Ac(pq)	0
asp	OAA(abcd)+Glu(efghi)	Asp(abcd)+AKG(efghi)	0
asn	Asp(abcd)+2ATP+NH4	Asn(abcd)	0
ala	Pyr(abc)+Glu(defgh)	Ala(abc)+AKG(defgh)	0
ser	G3P(abc)+Glu(defgh)	Ser(abc)+AKG(defgh)+NADH	0
gly1	Ser(abc)	Gly(ab)+MEETHF(c)	1
gldc	Gly(ab)	CO2(a)+MEETHF(b)+NADH+NH4	1
gly2	Thr(abcd)	Gly(ab)+AcCoA(cd)+NADH	0
cys	Ser(abc)+AcCoA(de)+3ATP+ 4NADPH+SO4	Cys(abc)+Ac(de)	0
lys1 [a]	Asp(abcd)+Pyr(efg)+Glu(hijkl)+ SucCoA(mnop)+ATP+2NADPH	LLDAP(abcdgfe)+AKG(hijkl)+ Suc(mnop,ponm)	0
lys2 [a]	LLDAP(abcdefg)	Lys(abcdef)+CO2(g)	0
thr	Asp(abcd)+2ATP+2NADPH	Thr(abcd)	0
met	Asp(abcd)+METHF(e)+Cys(fgh)+ SucCoA(ijkl)+ATP+2NADPH	Met(abcde)+Pyr(fgh)+ Suc(ijkl,lkji)+NH4	0
val	Pyr(abc)+Pyr(def)+Glu(ghijk)+ NADPH	Val(abcef)+CO2(d)+AKG(ghijk)	0
leu	AcCoA(ab)+Pyr(cde)+Pyr(fgh)+ Glu(ijklm)+NADPH	Leu(abdghe)+CO2(c)+CO2(f)+ AKG(ijklm)+NADH	0
ile	Thr(abcd)+Pyr(efg)+Glu(hijkl)+ NADPH	Ile(abfcdg)+CO2(e)+AKG(hijkl)+ NH4	0
phe	PEP(abc)+PEP(def)+E4P(ghij)+ Glu(klmno)+ATP+NADPH	Phe(abcefghij)+CO2(d)+ AKG(klmno)	0
tyr	PEP(abc)+PEP(def)+E4P(ghij)+ Glu(klmno)+ATP+NADPH	Tyr(abcefghij)+CO2(d)+ AKG(klmno)+NADH	0
trp	Ser(abc)+R5P(defgh)+PEP(ijk)+ E4P(lmno)+PEP(pqr)+Gln(stuvw) +3ATP+NADPH	Trp(abcedklmnoj)+CO2(i)+ GAP(fgh)+Pyr(pqr)+Glu(stuvw)	0
his	R5P(abcde)+FTHF(f)+Gln(ghijk)+ Asp(lmno)+5ATP	His(edcbaf)+AKG(ghijk)+ Fum(lmno)+2NADH	0
#One-carbon metabolism			
ocm1	MEETHF(a)+NADH	METHF(a)	0
ocm2	MEETHF(a)	FTHF(a)+NADPH	0
#Oxidative phosphorylation [b]			

atps1	NADH+0.5O2	3(2)ATP	0
atps2	FADH+0.5O2	2(1)ATP	0
#Transhydrogenation			
nnt	NADH	NADPH	1
#ATP hydrolysis			
atpm	ATP	ATP.ex	0
#Transportation			
glk [c]	Glc.ex(abcdef)+ATP	G6P(abcdef)	0
glcpts [c]	Glc.ex(abcdef)+PEP(ghi)	G6P(abcdef)+Pyr(ghi)	0
co2out	CO2(a)	CO2.ex(a)	0
o2in	O2.ex	O2	0
acout	Ac(ab)	Ac.ex(ab)	0
nh4in	NH4.ex	NH4	0
so4in	SO4.ex	SO4	0
#Biomass formation			
biom	0.488Ala+0.281Arg+0.229Asn+ 0.229Asp+0.087Cys+0.250Glu+ 0.250Gln+0.582Gly+0.090His+ 0.276Ile+0.428Leu+0.326Lys+ 0.146Met+0.176Phe+0.210Pro+ 0.205Ser+0.241Thr+0.054Trp+ 0.131Tyr+0.402Val+0.205G6P+ 0.071F6P+0.754R5P+0.129GAP+ 0.619G3P+0.051PEP+0.083Pyr+ 2.510AcCoA+0.087AKG+ 0.340OAA+0.443MEETHF+ 33.247ATP+5.363NADPH	39.68Biomass+1.455NADH	0
#CO_2 dilution [d]			
co2_d	0CO2.u(a)	0CO2(a)	0
#Amino acid dilution [d]			
ala_d1	0Ala(abc)	Ala.s(abc)	0
ala_d2	0Ala.u(abc)	Ala.s(abc)	0
ala_d3	Ala.s(abc)	sink	0
gly_d1	0Gly(ab)	Gly.s(ab)	0
gly_d2	0Gly.u(ab)	Gly.s(ab)	0
gly_d3	Gly.s(ab)	sink	0
val_d1	0Val(abcde)	Val.s(abcde)	0
val_d2	0Val.u(abcde)	Val.s(abcde)	0
val_d3	Val.s(abcde)	sink	0
leu_d1	0Leu(abcdef)	Leu.s(abcdef)	0
leu_d2	0Leu.u(abcdef)	Leu.s(abcdef)	0
leu_d3	Leu.s(abcdef)	sink	0
ile_d1	0Ile(abcdef)	Ile.s(abcdef)	0
ile_d2	0Ile.u(abcdef)	Ile.s(abcdef)	0

ile_d3	Ile.s(abcdef)	sink	0
ser_d1	0Ser(abc)	Ser.s(abc)	0
ser_d2	0Ser.u(abc)	Ser.s(abc)	0
ser_d3	Ser.s(abc)	sink	0
phe_d1	0Phe(abcdefghi)	Phe.s(abcdefghi)	0
phe_d2	0Phe.u(abcdefghi)	Phe.s(abcdefghi)	0
phe_d3	Phe.s(abcdefghi)	sink	0
asp_d1	0Asp(abcd)	Asp.s(abcd)	0
asp_d2	0Asp.u(abcd)	Asp.s(abcd)	0
asp_d3	Asp.s(abcd)	sink	0
glu_d1	0Glu(abcde)	Glu.s(abcde)	0
glu_d2	0Glu.u(abcde)	Glu.s(abcde)	0
glu_d3	Glu.s(abcde)	sink	0
tyr_d1	0Tyr(abcdefghi)	Tyr.s(abcdefghi)	0
tyr_d2	0Tyr.u(abcdefghi)	Tyr.s(abcdefghi)	0
tyr_d3	Tyr.s(abcdefghi)	sink	0

[a] The two reactions for lysine biosynthesis can be merged into:

Asp(abcd)+Pyr(efg)+Glu(hijkl)+SucCoA(mnop)+ATP+2NADPH -> Lys(abcdgf)+CO2(e)+AKG(hijkl)+

Suc(mnop,ponm)

[b] The P/O ratios of NADH and FADH were set 3 and 2, respectively, for flux estimation using synthetic data. For flux estimation using experimental data, the values were set to 2 and 1, respectively.

[c] The reaction by glucose kinase (glk) was used in the model with synthetic data, whereas glucose phosphotransferase system (glcpts) was used for flux estimation with experimental data.

[d] Dilution reactions were included only in the model using experimental data. The suffix ".s" denotes the sampling pool of a metabolite, and ".u" denotes the unlabeled pool. Pseudo metabolite "sink" was not balanced as an end metabolite of the network. A zero coefficient before a metabolite indicates that current reaction makes no contribution to the mass balance of the metabolite node, but it is still involved in the EMU network decomposition to account for the dilution effect.

Table S2. Synthetic MDVs for flux estimation of the *E. coli* model. Fragment IDs are represented as EMU, i.e., “metabolite” + “_” + “carbon number”. The MDVs are simulated with the labeling strategy of addition of a mixture of 75% [1-^{13}C] glucose and 25% [U-^{13}C] glucose.

Fragment ID	Mean	SD
Ala_23	0.511,0.285,0.204	0.010,0.010,0.010
Ala_123	0.297,0.385,0.186,0.131	0.010,0.010,0.010,0.010
Gly_2	0.662,0.338	0.010,0.010
Gly_12	0.497,0.325,0.178	0.010,0.010,0.010
Val_2345	0.261,0.291,0.290,0.116,0.042	0.010,0.010,0.010,0.010,0.010
Val_12345	0.152,0.281,0.266,0.199,0.076,0.027	0.010,0.010,0.010,0.010,0.010,0.010
Leu_23456	0.165,0.280,0.290,0.180,0.069,0.015	0.010,0.010,0.010,0.010,0.010,0.010
Ile_23456	0.154,0.272,0.288,0.191,0.076,0.019	0.010,0.010,0.010,0.010,0.010,0.010
Ser_23	0.430,0.450,0.119	0.010,0.010,0.010
Ser_123	0.324,0.384,0.229,0.063	0.010,0.010,0.010,0.010
Thr_234	0.302,0.363,0.240,0.095	0.010,0.010,0.010,0.010
Thr_1234	0.231,0.334,0.230,0.150,0.055	0.010,0.010,0.010,0.010,0.010
Phe_12	0.542,0.273,0.186	0.010,0.010,0.010
Phe_23456789	0.105,0.183,0.223,0.185,0.140,0.090,0.051,0.019,0.005	0.010,0.010,0.010,0.010,0.010,0.010,0.010,0.010,0.010
Phe_123456789	0.085,0.161,0.193,0.190,0.154,0.108,0.063,0.031,0.012,0.003	0.010,0.010,0.010,0.010,0.010,0.010,0.010,0.010,0.010,0.010
Asp_12	0.504,0.314,0.182	0.010,0.010,0.010
Asp_234	0.302,0.363,0.240,0.095	0.010,0.010,0.010,0.010
Asp_1234	0.231,0.334,0.230,0.150,0.055	0.010,0.010,0.010,0.010,0.010
Glu_2345	0.191,0.341,0.285,0.148,0.035	0.010,0.010,0.010,0.010,0.010
Glu_12345	0.151,0.272,0.289,0.192,0.077,0.019	0.010,0.010,0.010,0.010,0.010,0.010
Tyr_12	0.542,0.273,0.186	0.010,0.010,0.010

Table S3. Synthetic exchange flux and growth rate for flux estimation of the *E. coli* model.

**Reaction ID**	**Mean (mmol gCDW^{-1} h^{-1})**	**SD**
glk	10	1
biom	0.7	0.1

Table S4. Experimental MDVs for flux estimation of the *E. coli* model. Fragment IDs are represented as EMU, i.e., “metabolite” + “_” + “carbon number”. Data was corrected for the natural abundance of isotopomers. Amino acids were added with suffix “.s” to denote the sampling pools since dilution effect of unlabeled pools of these metabolites were considered (Table S1). The MDVs are measured with the labeling strategy of addition of a mixture of 77% [1-^{13}C] glucose and 20.5% [U-^{13}C] glucose.

Fragment ID	Mean	SD
Ala.s_23	0.4791,0.3211,0.1998	0.004,0.004,0.004
Ala.s _123	0.4699,0.3207,0.0438,0.1657	0.004,0.004,0.004,0.004
Gly.s _2	0.7844,0.2156	0.004,0.004
Val.s _2345	0.2340,0.2999,0.2920,0.1315,0.0426	0.004,0.004,0.004,0.004,0.004
Val.s _12345	0.2387,0.3053,0.2080,0.1532,0.0608,0.0341	0.004,0.004,0.004,0.004,0.004,0.004
Leu.s _23456	0.1384,0.2660,0.2934,0.2022,0.0801,0.0199	0.004,0.004,0.004,0.004,0.004,0.004
Ile.s _23456	0.1735,0.2767,0.2773,0.1816,0.0711,0.0197	0.004,0.004,0.004,0.004,0.004,0.004
Ser.s _23	0.4580,0.3719,0.1701	0.004,0.004,0.004
Phe.s _12	0.7661,0.0509,0.1829	0.004,0.004,0.004
Phe.s _23456789	0.1377,0.2086,0.2296,0.1633,0.1124,0.0740,0.0457,0.0211,0.0077	0.004,0.004,0.004,0.004,0.004,0.004,0.004,0.004,0.004
Asp.s _234	0.3494,0.3624,0.1960,0.0922	0.004,0.004,0.004,0.004
Asp.s _1234	0.3024,0.3440,0.1788,0.1319,0.0429	0.004,0.004,0.004,0.004,0.004
Glu.s _12345	0.1796,0.2757,0.2778,0.1791,0.0682,0.0197	0.004,0.004,0.004,0.004,0.004,0.004
Tyr.s _12	0.7616,0.0516,0.1869	0.004,0.004,0.004

Table S5. Experimental exchange fluxes for flux estimation of the *E. coli* model.

Reaction ID	Mean (mmol gCDW^{-1} h^{-1})	SD
glcpts	11.59	1
acout	8.11	0.6

Table S6. Metabolic reactions with atom mapping in the *Synechocystis* model.

Reaction ID	Reactant IDs(atoms)	Product IDs(atoms)	Reversibility
#Glycolysis and OPP pathway			
pgi	G6P(abcdef)	F6P(abcdef)	1
g6pdh	G6P(abcdef)	Ru5P(bcdef)+CO2(a)	0
pfk	F6P(abcdef)	FBP(abcdef)	1
fba	FBP(abcdef)	DHAP(cba)+GAP(def)	1
tpi	DHAP(abc)	GAP(abc)	1
gapdh	GAP(abc)	G3P(abc)	1
gpm	G3P(abc)	G2P(abc)	1
eno	G2P(abc)	PEP(abc)	1
pk	PEP(abc)	Pyr(abc)	1
#CBB cycle			
rpe	Ru5P(abcde)	X5P(abcde)	1
rpi	Ru5P(abcde)	R5P(abcde)	1
prk	Ru5P(abcde)	RuBP(abcde)	0
rbc1	RuBP(abcde)+CO2(f)	G3P(cde)+G3P(fba)	0
tkt1	X5P(abcde)	TK(ab)+GAP(cde)	1
tkt2	F6P(abcdef)	TK(ab)+E4P(cdef)	1
tkt3	S7P(abcdefg)	TK(ab)+R5P(cdefg)	1
tal1	F6P(abcdef)	TA(abc)+GAP(def)	1
tal2	S7P(abcdefg)	TA(abc)+E4P(defg)	1
sba	DHAP(abc)+E4P(defg)	SBP(cbadefg)	0
sbp	SBP(abcdefg)	S7P(abcdefg)	0
#TCA cycle			
pdh	Pyr(abc)	AcCoA(bc)+CO2(a)	0
cs	OAA(abcd)+AcCoA(ef)	Cit(dcbfea)	0
can	Cit(abcdef)	ICit(abcdef)	1
icd	ICit(abcdef)	AKG(abcde)+CO2(f)	1
sdh	Suc(abcd,dcba)	Fum(abcd,dcba)	1
fum	Fum(abcd,dcba)	Mal(abcd)	1
mdh	Mal(abcd)	OAA(abcd)	1
#Glyoxylate Shunt			
icl	ICit(abcdef)	Glx(ed)+Suc(abcf)	0
ms	Glx(ab)+AcCoA(cd)	Mal(abdc)	1
#Amphibolic reactions			
me	Mal(abcd)	Pyr(abc)+CO2(d)	0
ppc	PEP(abc)+CO2(d)	OAA(abcd)	0
#Photorespiration			
rbc2	RuBP(abcde)	G3P(cde)+PG(ba)	0
pgp	PG(ab)	Gc(ab)	0
gld	Gc(ab)	Glx(ab)	0

gt	Glx(ab)+Glx(cd)	Ga(abc)+CO2(d)	0
glyk	Ga(abc)	G3P(abc)	1
#Transportation			
co2in	CO2.ex(a)	CO2(a)	0
#Biomass formation			
biom	0.715R5P+3.624AcCoA+1.191G6P+ 0.501E4P+1.205G3P+1.002PEP+ 1.197Pyr+2.039OAA+1.233AKG+ 0.133 GAP	Biomass+0.683Fum+1.017CO2	0
#Dilution [a]			
dhap_d1	0DHAP(abc)	DHAP.s(abc)	0
dhap_d2	0DHAP.u(abc)	DHAP.s(abc)	0
dhap_d3	DHAP.s(abc)	sink	0
f6p_d1	0F6P(abcdef)	F6P.s(abcdef)	0
f6p_d2	0F6P.u(abcdef)	F6P.s(abcdef)	0
f6p_d3	F6P.s(abc)	sink	0
gap_d1	0GAP(abc)	GAP.s(abc)	0
gap_d2	0GAP.u(abc)	GAP.s(abc)	0
gap_d3	GAP.s(abc)	sink	0
r5p_d1	0R5P(abcde)	R5P.s(abcde)	0
r5p_d2	0R5P.u(abcde)	R5P.s(abcde)	0
r5p_d3	R5P.s(abcde)	sink	0

[a] Dilution reactions were included only in the model using experimental data. The suffix ".s" denotes the sampling pool of a metabolite, and ".u" denotes the unlabeled pool. Pseudo metabolite "sink" was not balanced as an end metabolite of the network. Zero coefficient before a metabolite indicates that current reaction makes no contribution to the mass balance of the metabolite node, but it is still involved in the EMU network decomposition to account for the dilution effect.

Table S7. Synthetic MDVs for flux estimation of the *Synechocystis* model. Fragment IDs are represented as EMU, i.e., "metabolite" + "_" + "carbon number". The MDVs are simulated with the labeling strategy of addition of 50% $^{13}CO_2$ and a constant concentration of 10 µmol $gCDW^{-1}$ for all metabolites.

Fragment ID	Time (s)	Mean	SD
GAP_123	10	0.853,0.140,0.006,0.000	0.010,0.010,0.010,0.010
GAP_123	30	0.626,0.325,0.046,0.004	0.010,0.010,0.010,0.010
GAP_123	60	0.447,0.412,0.124,0.017	0.010,0.010,0.010,0.010
GAP_123	120	0.275,0.435,0.241,0.049	0.010,0.010,0.010,0.010
GAP_123	240	0.166,0.404,0.335,0.094	0.010,0.010,0.010,0.010
GAP_123	360	0.129,0.378,0.371,0.122	0.010,0.010,0.010,0.010
Ga_12	10	0.876,0.121,0.003	0.010,0.010,0.010
Ga_12	30	0.658,0.315,0.027	0.010,0.010,0.010
Ga_12	60	0.511,0.413,0.076	0.010,0.010,0.010
Ga_12	120	0.380,0.473,0.147	0.010,0.010,0.010
Ga_12	240	0.288,0.496,0.216	0.010,0.010,0.010
Ga_12	360	0.252,0.500,0.248	0.010,0.010,0.010
Ga_123	10	0.864,0.130,0.006,0.000	0.010,0.010,0.010,0.010
Ga_123	30	0.629,0.323,0.044,0.004	0.010,0.010,0.010,0.010
Ga_123	60	0.450,0.411,0.123,0.016	0.010,0.010,0.010,0.010
Ga_123	120	0.277,0.435,0.240,0.049	0.010,0.010,0.010,0.010
Ga_123	240	0.167,0.405,0.334,0.094	0.010,0.010,0.010,0.010
Ga_123	360	0.129,0.378,0.371,0.122	0.010,0.010,0.010,0.010
PEP_123	10	0.892,0.104,0.004,0.000	0.010,0.010,0.010,0.010
PEP_123	30	0.665,0.294,0.038,0.003	0.010,0.010,0.010,0.010
PEP_123	60	0.478,0.395,0.112,0.015	0.010,0.010,0.010,0.010
PEP_123	120	0.294,0.431,0.230,0.046	0.010,0.010,0.010,0.010
PEP_123	240	0.173,0.406,0.330,0.092	0.010,0.010,0.010,0.010
PEP_123	360	0.130,0.379,0.370,0.121	0.010,0.010,0.010,0.010
G3P_23	10	0.967,0.033,0.001	0.010,0.010,0.010

G3P_23	30	0.867,0.122,0.011	0.010,0.010,0.010
G3P_23	60	0.684,0.270,0.046	0.010,0.010,0.010
G3P_23	120	0.462,0.422,0.117	0.010,0.010,0.010
G3P_23	240	0.310,0.490,0.200	0.010,0.010,0.010
G3P_23	360	0.256,0.500,0.245	0.010,0.010,0.010
G3P_123	10	0.820,0.172,0.008,0.000	0.010,0.010,0.010,0.010
G3P_123	30	0.609,0.337,0.050,0.004	0.010,0.010,0.010,0.010
G3P_123	60	0.437,0.415,0.130,0.018	0.010,0.010,0.010,0.010
G3P_123	120	0.270,0.435,0.245,0.050	0.010,0.010,0.010,0.010
G3P_123	240	0.165,0.404,0.336,0.095	0.010,0.010,0.010,0.010
G3P_123	360	0.128,0.378,0.372,0.122	0.010,0.010,0.010,0.010
DHAP_123	10	0.862,0.132,0.006,0.000	0.010,0.010,0.010,0.010
DHAP_123	30	0.630,0.322,0.044,0.004	0.010,0.010,0.010,0.010
DHAP_123	60	0.449,0.411,0.123,0.017	0.010,0.010,0.010,0.010
DHAP_123	120	0.276,0.435,0.240,0.049	0.010,0.010,0.010,0.010
DHAP_123	240	0.167,0.404,0.335,0.094	0.010,0.010,0.010,0.010
DHAP_123	360	0.129,0.378,0.371,0.122	0.010,0.010,0.010,0.010
Mal_234	10	0.929,0.069,0.001,0.000	0.010,0.010,0.010,0.010
Mal_234	30	0.851,0.141,0.008,0.000	0.010,0.010,0.010,0.010
Mal_234	60	0.703,0.255,0.040,0.003	0.010,0.010,0.010,0.010
Mal_234	120	0.440,0.398,0.143,0.019	0.010,0.010,0.010,0.010
Mal_234	240	0.220,0.423,0.289,0.069	0.010,0.010,0.010,0.010
Mal_234	360	0.138,0.385,0.363,0.115	0.010,0.010,0.010,0.010
Suc_1234	10	0.949,0.050,0.001,0.000,0.000	0.010,0.010,0.010,0.010,0.010
Suc_1234	30	0.861,0.126,0.012,0.001,0.000	0.010,0.010,0.010,0.010,0.010
Suc_1234	60	0.681,0.257,0.055,0.007,0.001	0.010,0.010,0.010,0.010,0.010
Suc_1234	120	0.391,0.377,0.180,0.047,0.006	0.010,0.010,0.010,0.010,0.010
Suc_1234	240	0.159,0.346,0.322,0.146,0.027	0.010,0.010,0.010,0.010,0.010
Suc_1234	360	0.077,0.271,0.369,0.228,0.054	0.010,0.010,0.010,0.010,0.010
Fum_1234	10	0.942,0.056,0.002,0.000,0.000	0.010,0.010,0.010,0.010,0.010
Fum_1234	30	0.843,0.141,0.015,0.001,0.000	0.010,0.010,0.010,0.010,0.010

Fum_1234	60	0.658,0.271,0.062,0.009,0.001	0.010,0.010,0.010,0.010,0.010
Fum_1234	120	0.373,0.381,0.190,0.050,0.006	0.010,0.010,0.010,0.010,0.010
Fum_1234	240	0.152,0.343,0.326,0.151,0.028	0.010,0.010,0.010,0.010,0.010
Fum_1234	360	0.076,0.270,0.370,0.230,0.055	0.010,0.010,0.010,0.010,0.010
Ru5P_12345	10	0.833,0.153,0.013,0.001,0.000,0.000	0.010,0.010,0.010,0.010,0.010,0.010
Ru5P_12345	30	0.526,0.354,0.102,0.017,0.002,0.000	0.010,0.010,0.010,0.010,0.010,0.010
Ru5P_12345	60	0.289,0.397,0.229,0.072,0.012,0.001	0.010,0.010,0.010,0.010,0.010,0.010
Ru5P_12345	120	0.123,0.311,0.328,0.180,0.052,0.006	0.010,0.010,0.010,0.010,0.010,0.010
Ru5P_12345	240	0.051,0.205,0.334,0.275,0.115,0.019	0.010,0.010,0.010,0.010,0.010,0.010
Ru5P_12345	360	0.033,0.161,0.315,0.309,0.152,0.030	0.010,0.010,0.010,0.010,0.010,0.010
R5P_12345	10	0.797,0.181,0.021,0.001,0.000,0.000	0.010,0.010,0.010,0.010,0.010,0.010
R5P_12345	30	0.449,0.377,0.142,0.028,0.003,0.000	0.010,0.010,0.010,0.010,0.010,0.010
R5P_12345	60	0.240,0.387,0.261,0.092,0.017,0.002	0.010,0.010,0.010,0.010,0.010,0.010
R5P_12345	120	0.109,0.297,0.334,0.194,0.059,0.007	0.010,0.010,0.010,0.010,0.010,0.010
R5P_12345	240	0.049,0.200,0.333,0.279,0.119,0.020	0.010,0.010,0.010,0.010,0.010,0.010
R5P_12345	360	0.033,0.160,0.315,0.310,0.153,0.030	0.010,0.010,0.010,0.010,0.010,0.010
RuBP_12345	10	0.843,0.144,0.012,0.001,0.000,0.000	0.010,0.010,0.010,0.010,0.010,0.010
RuBP_12345	30	0.536,0.348,0.098,0.016,0.002,0.000	0.010,0.010,0.010,0.010,0.010,0.010
RuBP_12345	60	0.294,0.396,0.226,0.071,0.012,0.001	0.010,0.010,0.010,0.010,0.010,0.010
RuBP_12345	120	0.125,0.312,0.327,0.179,0.051,0.006	0.010,0.010,0.010,0.010,0.010,0.010
RuBP_12345	240	0.052,0.206,0.334,0.275,0.114,0.019	0.010,0.010,0.010,0.010,0.010,0.010
RuBP_12345	360	0.033,0.161,0.315,0.309,0.152,0.030	0.010,0.010,0.010,0.010,0.010,0.010
G6P_123456	10	0.818,0.165,0.016,0.001,0.000,0.000,0.000	0.010,0.010,0.010,0.010,0.010,0.010,0.010
G6P_123456	30	0.473,0.374,0.127,0.024,0.003,0.000,0.000	0.010,0.010,0.010,0.010,0.010,0.010,0.010
G6P_123456	60	0.232,0.378,0.261,0.101,0.024,0.003,0.000	0.010,0.010,0.010,0.010,0.010,0.010,0.010
G6P_123456	120	0.085,0.250,0.320,0.227,0.094,0.022,0.002	0.010,0.010,0.010,0.010,0.010,0.010,0.010
G6P_123456	240	0.029,0.138,0.277,0.300,0.185,0.062,0.009	0.010,0.010,0.010,0.010,0.010,0.010,0.010
G6P_123456	360	0.017,0.098,0.239,0.312,0.230,0.090,0.015	0.010,0.010,0.010,0.010,0.010,0.010,0.010
F6P_123456	10	0.775,0.203,0.021,0.001,0.000,0.000,0.000	0.010,0.010,0.010,0.010,0.010,0.010,0.010
F6P_123456	30	0.426,0.400,0.143,0.027,0.003,0.000,0.000	0.010,0.010,0.010,0.010,0.010,0.010,0.010
F6P_123456	60	0.213,0.376,0.273,0.108,0.026,0.003,0.000	0.010,0.010,0.010,0.010,0.010,0.010,0.010

F6P_123456	120	0.079,0.244,0.322,0.233,0.097,0.022,0.002	0.010,0.010,0.010,0.010,0.010,0.010,0.010
F6P_123456	240	0.028,0.135,0.276,0.302,0.187,0.063,0.009	0.010,0.010,0.010,0.010,0.010,0.010,0.010
F6P_123456	360	0.017,0.097,0.239,0.312,0.230,0.090,0.015	0.010,0.010,0.010,0.010,0.010,0.010,0.010
Cit_12345	10	0.934,0.064,0.002,0.000,0.000,0.000	0.010,0.010,0.010,0.010,0.010,0.010
Cit_12345	30	0.858,0.130,0.012,0.001,0.000,0.000	0.010,0.010,0.010,0.010,0.010,0.010
Cit_12345	60	0.687,0.248,0.056,0.008,0.001,0.000	0.010,0.010,0.010,0.010,0.010,0.010
Cit_12345	120	0.368,0.354,0.198,0.067,0.013,0.001	0.010,0.010,0.010,0.010,0.010,0.010
Cit_12345	240	0.114,0.277,0.322,0.207,0.071,0.010	0.010,0.010,0.010,0.010,0.010,0.010
Cit_12345	360	0.041,0.179,0.322,0.295,0.137,0.026	0.010,0.010,0.010,0.010,0.010,0.010
Cit_123456	10	0.922,0.074,0.003,0.000,0.000,0.000,0.000	0.010,0.010,0.010,0.010,0.010,0.010,0.010
Cit_123456	30	0.824,0.152,0.021,0.002,0.000,0.000,0.000	0.010,0.010,0.010,0.010,0.010,0.010,0.010
Cit_123456	60	0.615,0.278,0.085,0.019,0.003,0.000,0.000	0.010,0.010,0.010,0.010,0.010,0.010,0.010
Cit_123456	120	0.288,0.338,0.232,0.106,0.031,0.005,0.000	0.010,0.010,0.010,0.010,0.010,0.010,0.010
Cit_123456	240	0.075,0.211,0.297,0.250,0.127,0.036,0.005	0.010,0.010,0.010,0.010,0.010,0.010,0.010
Cit_123456	360	0.022,0.114,0.253,0.306,0.212,0.080,0.013	0.010,0.010,0.010,0.010,0.010,0.010,0.010
S7P_1234567	10	0.740,0.226,0.032,0.002,0.000,0.000,0.000,0.000	0.010,0.010,0.010,0.010,0.010,0.010,0.010,0.010
S7P_1234567	30	0.345,0.398,0.196,0.052,0.009,0.001,0.000,0.000	0.010,0.010,0.010,0.010,0.010,0.010,0.010,0.010
S7P_1234567	60	0.148,0.324,0.304,0.160,0.052,0.011,0.001,0.000	0.010,0.010,0.010,0.010,0.010,0.010,0.010,0.010
S7P_1234567	120	0.048,0.179,0.291,0.268,0.151,0.052,0.010,0.001	0.010,0.010,0.010,0.010,0.010,0.010,0.010,0.010
S7P_1234567	240	0.015,0.085,0.210,0.289,0.241,0.121,0.034,0.004	0.010,0.010,0.010,0.010,0.010,0.010,0.010,0.010
S7P_1234567	360	0.008,0.057,0.168,0.276,0.271,0.160,0.053,0.007	0.010,0.010,0.010,0.010,0.010,0.010,0.010,0.010

Table S8. Synthetic exchange flux for flux estimation of the *Synechocystis* model.

Reaction ID	Mean (μmol gCDW^{-1} s^{-1})	SD
co2in	10	1

Table S9. Experimental MDVs for flux estimation of the *Synechocystis* model. Fragment IDs are represented as EMU, i.e., “metabolite” + “_” + “carbon number”. Data was corrected for the natural abundance of isotopomers. DHAP, F6P, GAP and R5P were added with suffix “.s” to denote the sampled pools since dilution effect of unlabeled pools of these metabolites were considered (Table S6). The MDVs are measured with the labeling strategy of addition of 50% $NaH^{13}CO_3$.

Fragment ID	Time (s)	Mean	SD
G3P_123	20	0.6405,0.3306,0.0237,0.0054	0.0050,0.0050,0.0050,0.0050
G3P_123	40	0.5249,0.3885,0.0764,0.0104	0.0050,0.0050,0.0050,0.0050
G3P_123	60	0.4333,0.4198,0.1206,0.0264	0.0050,0.0050,0.0050,0.0050
G3P_123	90	0.3741,0.4147,0.1691,0.0423	0.0050,0.0050,0.0050,0.0050
G3P_123	130	0.3075,0.3987,0.2312,0.0627	0.0050,0.0050,0.0050,0.0050
G3P_123	250	0.2230,0.3740,0.3019,0.1012	0.0050,0.0050,0.0050,0.0050
G3P_123	480	0.1837,0.3618,0.3401,0.1144	0.0050,0.0050,0.0050,0.0050
G3P_123	610	0.1920,0.3539,0.3360,0.1182	0.0050,0.0050,0.0050,0.0050
DHAP.s_123	20	0.7340,0.2484,0.0176,0.0000	0.0130,0.0130,0.0130,0.0130
DHAP.s_123	40	0.6179,0.3253,0.0568,0.0000	0.0130,0.0130,0.0130,0.0130
DHAP.s_123	60	0.5398,0.3599,0.0862,0.0141	0.0130,0.0130,0.0130,0.0130
DHAP.s_123	90	0.4391,0.4040,0.1229,0.0341	0.0130,0.0130,0.0130,0.0130
DHAP.s_123	130	0.3515,0.3815,0.2060,0.0610	0.0130,0.0130,0.0130,0.0130
DHAP.s_123	250	0.2614,0.3643,0.2787,0.0956	0.0130,0.0130,0.0130,0.0130
DHAP.s_123	480	0.2114,0.3445,0.3326,0.1115	0.0130,0.0130,0.0130,0.0130
DHAP.s_123	610	0.2128,0.3451,0.3355,0.1066	0.0130,0.0130,0.0130,0.0130
F6P.s_123456	20	0.5992,0.3321,0.0597,0.0062,0.0017,0.0010,0.0000	0.0170,0.0170,0.0170,0.0170,0.0170,0.0170,0.0170
F6P.s_123456	40	0.4484,0.3498,0.1498,0.0411,0.0087,0.0021,0.0000	0.0170,0.0170,0.0170,0.0170,0.0170,0.0170,0.0170
F6P.s_123456	60	0.3026,0.3403,0.2484,0.0817,0.0216,0.0047,0.0007	0.0170,0.0170,0.0170,0.0170,0.0170,0.0170,0.0170
F6P.s_123456	130	0.1690,0.2395,0.2818,0.1950,0.0876,0.0240,0.0032	0.0170,0.0170,0.0170,0.0170,0.0170,0.0170,0.0170
F6P.s_123456	250	0.1123,0.1531,0.2653,0.2489,0.1579,0.0550,0.0074	0.0170,0.0170,0.0170,0.0170,0.0170,0.0170,0.0170
F6P.s_123456	480	0.1367,0.1180,0.2271,0.2556,0.1805,0.0705,0.0116	0.0170,0.0170,0.0170,0.0170,0.0170,0.0170,0.0170
F6P.s_123456	610	0.0917,0.1276,0.2279,0.2701,0.1937,0.0758,0.0133	0.0170,0.0170,0.0170,0.0170,0.0170,0.0170,0.0170

G6P_123456	20	0.6019,0.3011,0.0892,0.0078,0.0000,0.0000,0.0000	0.0140,0.0140,0.0140,0.0140,0.0140,0.0140,0.0140
G6P_123456	40	0.4340,0.3366,0.1826,0.0393,0.0065,0.0010,0.0000	0.0140,0.0140,0.0140,0.0140,0.0140,0.0140,0.0140
G6P_123456	60	0.3427,0.3236,0.2348,0.0766,0.0194,0.0029,0.0000	0.0140,0.0140,0.0140,0.0140,0.0140,0.0140,0.0140
G6P_123456	130	0.2124,0.2263,0.2866,0.1737,0.0776,0.0209,0.0026	0.0140,0.0140,0.0140,0.0140,0.0140,0.0140,0.0140
G6P_123456	250	0.0977,0.1517,0.2680,0.2548,0.1612,0.0580,0.0084	0.0140,0.0140,0.0140,0.0140,0.0140,0.0140,0.0140
G6P_123456	480	0.1129,0.1195,0.2448,0.2545,0.1825,0.0741,0.0117	0.0140,0.0140,0.0140,0.0140,0.0140,0.0140,0.0140
G6P_123456	610	0.0884,0.1217,0.2444,0.2638,0.1929,0.0769,0.0119	0.0140,0.0140,0.0140,0.0140,0.0140,0.0140,0.0140
GAP.s_123	20	0.7421,0.2579,0.0000,0.0000	0.0130,0.0130,0.0130,0.0130
GAP.s_123	40	0.6078,0.3170,0.0716,0.0036	0.0130,0.0130,0.0130,0.0130
GAP.s_123	60	0.5376,0.3825,0.0710,0.0088	0.0130,0.0130,0.0130,0.0130
GAP.s_123	130	0.3722,0.3861,0.1941,0.0475	0.0130,0.0130,0.0130,0.0130
GAP.s_123	250	0.2477,0.3627,0.3092,0.0805	0.0130,0.0130,0.0130,0.0130
GAP.s_123	480	0.2444,0.3355,0.3173,0.1028	0.0130,0.0130,0.0130,0.0130
GAP.s_123	610	0.2515,0.3406,0.3158,0.0921	0.0130,0.0130,0.0130,0.0130
PEP_123	20	0.6393,0.3353,0.0251,0.0003	0.0050,0.0050,0.0050,0.0050
PEP_123	40	0.4989,0.4159,0.0755,0.0096	0.0050,0.0050,0.0050,0.0050
PEP_123	60	0.4112,0.4396,0.1252,0.0240	0.0050,0.0050,0.0050,0.0050
PEP_123	90	0.3392,0.4359,0.1804,0.0446	0.0050,0.0050,0.0050,0.0050
PEP_123	130	0.2786,0.4236,0.2322,0.0655	0.0050,0.0050,0.0050,0.0050
PEP_123	250	0.1881,0.3880,0.3191,0.1049	0.0050,0.0050,0.0050,0.0050
PEP_123	480	0.1592,0.3696,0.3491,0.1220	0.0050,0.0050,0.0050,0.0050
PEP_123	610	0.1663,0.3678,0.3577,0.1082	0.0050,0.0050,0.0050,0.0050
R5P.s_12345	20	0.5999,0.3117,0.0776,0.0108,0.0000,0.0000	0.0150,0.0150,0.0150,0.0150,0.0150,0.0150
R5P.s_12345	40	0.4243,0.3509,0.1790,0.0393,0.0059,0.0006	0.0150,0.0150,0.0150,0.0150,0.0150,0.0150
R5P.s_12345	60	0.3334,0.3563,0.2323,0.0650,0.0117,0.0013	0.0150,0.0150,0.0150,0.0150,0.0150,0.0150
R5P.s_12345	90	0.2506,0.3336,0.2720,0.1181,0.0216,0.0042	0.0150,0.0150,0.0150,0.0150,0.0150,0.0150
R5P.s_12345	130	0.2076,0.2844,0.3065,0.1453,0.0474,0.0089	0.0150,0.0150,0.0150,0.0150,0.0150,0.0150
R5P.s_12345	250	0.1473,0.2197,0.3152,0.2095,0.0888,0.0195	0.0150,0.0150,0.0150,0.0150,0.0150,0.0150
R5P.s_12345	480	0.1357,0.1951,0.3099,0.2269,0.1068,0.0256	0.0150,0.0150,0.0150,0.0150,0.0150,0.0150
R5P.s_12345	610	0.1223,0.1906,0.3163,0.2347,0.1108,0.0252	0.0150,0.0150,0.0150,0.0150,0.0150,0.0150
RuBP_12345	20	0.5844,0.3448,0.0614,0.0083,0.0012,0.0000	0.0070,0.0070,0.0070,0.0070,0.0070,0.0070

RuBP_12345	40	0.4085,0.4008,0.1493,0.0361,0.0048,0.0006	0.0070,0.0070,0.0070,0.0070,0.0070,0.0070
RuBP_12345	60	0.3086,0.3988,0.2075,0.0720,0.0122,0.0009	0.0070,0.0070,0.0070,0.0070,0.0070,0.0070
RuBP_12345	90	0.2143,0.3594,0.2688,0.1218,0.0316,0.0041	0.0070,0.0070,0.0070,0.0070,0.0070,0.0070
RuBP_12345	130	0.1627,0.3169,0.2925,0.1656,0.0537,0.0086	0.0070,0.0070,0.0070,0.0070,0.0070,0.0070
RuBP_12345	250	0.1021,0.2405,0.3096,0.2335,0.0992,0.0150	0.0070,0.0070,0.0070,0.0070,0.0070,0.0070
RuBP_12345	480	0.0820,0.2039,0.3028,0.2639,0.1244,0.0230	0.0070,0.0070,0.0070,0.0070,0.0070,0.0070
RuBP_12345	610	0.0777,0.1956,0.3046,0.2723,0.1277,0.0222	0.0070,0.0070,0.0070,0.0070,0.0070,0.0070
S7P_1234567	20	0.4610,0.3745,0.1326,0.0277,0.0036,0.0007,0.0000,0.0000	0.0280,0.0280,0.0280,0.0280,0.0280,0.0280,0.0280,0.0280
S7P_1234567	40	0.2725,0.3701,0.2362,0.0943,0.0226,0.0038,0.0006,0.0000	0.0280,0.0280,0.0280,0.0280,0.0280,0.0280,0.0280,0.0280
S7P_1234567	60	0.1784,0.3225,0.2793,0.1532,0.0528,0.0120,0.0019,0.0000	0.0280,0.0280,0.0280,0.0280,0.0280,0.0280,0.0280,0.0280
S7P_1234567	90	0.1003,0.2545,0.2970,0.2160,0.0965,0.0302,0.0052,0.0004	0.0280,0.0280,0.0280,0.0280,0.0280,0.0280,0.0280,0.0280
S7P_1234567	130	0.0711,0.1957,0.2717,0.2479,0.1435,0.0561,0.0126,0.0015	0.0280,0.0280,0.0280,0.0280,0.0280,0.0280,0.0280,0.0280
S7P_1234567	250	0.0359,0.1157,0.2154,0.2680,0.2125,0.1129,0.0350,0.0047	0.0280,0.0280,0.0280,0.0280,0.0280,0.0280,0.0280,0.0280
S7P_1234567	480	0.0260,0.0917,0.1897,0.2655,0.2381,0.1371,0.0459,0.0060	0.0280,0.0280,0.0280,0.0280,0.0280,0.0280,0.0280,0.0280
S7P_1234567	610	0.0253,0.0946,0.1919,0.2630,0.2350,0.1374,0.0465,0.0062	0.0280,0.0280,0.0280,0.0280,0.0280,0.0280,0.0280,0.0280
Ru5P_12345	20	0.6011,0.3292,0.0645,0.0052,0.0000,0.0000	0.0130,0.0130,0.0130,0.0130,0.0130,0.0130
Ru5P_12345	40	0.4181,0.3930,0.1529,0.0321,0.0039,0.0000	0.0130,0.0130,0.0130,0.0130,0.0130,0.0130
Ru5P_12345	60	0.3132,0.3861,0.2230,0.0661,0.0110,0.0005	0.0130,0.0130,0.0130,0.0130,0.0130,0.0130
Ru5P_12345	90	0.2037,0.3739,0.2709,0.1222,0.0259,0.0035	0.0130,0.0130,0.0130,0.0130,0.0130,0.0130
Ru5P_12345	130	0.1539,0.3035,0.3194,0.1665,0.0501,0.0066	0.0130,0.0130,0.0130,0.0130,0.0130,0.0130
Ru5P_12345	250	0.0965,0.2240,0.3243,0.2383,0.0984,0.0186	0.0130,0.0130,0.0130,0.0130,0.0130,0.0130
Ru5P_12345	480	0.0713,0.1965,0.3235,0.2647,0.1208,0.0233	0.0130,0.0130,0.0130,0.0130,0.0130,0.0130
Ru5P_12345	610	0.0927,0.1855,0.3149,0.2629,0.1217,0.0224	0.0130,0.0130,0.0130,0.0130,0.0130,0.0130
Suc_1234	20	0.9640,0.0298,0.0037,0.0025,0.0000	0.0100,0.0100,0.0100,0.0100,0.0100
Suc_1234	40	0.9532,0.0416,0.0025,0.0019,0.0008	0.0100,0.0100,0.0100,0.0100,0.0100
Suc_1234	60	0.9433,0.0528,0.0039,0.0000,0.0000	0.0100,0.0100,0.0100,0.0100,0.0100
Suc_1234	90	0.9617,0.0331,0.0000,0.0052,0.0000	0.0100,0.0100,0.0100,0.0100,0.0100
Suc_1234	130	0.9389,0.0561,0.0036,0.0014,0.0000	0.0100,0.0100,0.0100,0.0100,0.0100
Suc_1234	250	0.9026,0.0688,0.0213,0.0073,0.0001	0.0100,0.0100,0.0100,0.0100,0.0100
Suc_1234	480	0.8336,0.0901,0.0494,0.0210,0.0059	0.0100,0.0100,0.0100,0.0100,0.0100
Suc_1234	610	0.7997,0.0975,0.0660,0.0272,0.0096	0.0100,0.0100,0.0100,0.0100,0.0100

Fum_1234	20	0.8762,0.1012,0.0183,0.0043,0.0000	0.0100,0.0100,0.0100,0.0100,0.0100
Fum_1234	40	0.8551,0.1008,0.0440,0.0001,0.0000	0.0100,0.0100,0.0100,0.0100,0.0100
Fum_1234	60	0.8334,0.1030,0.0636,0.0000,0.0000	0.0100,0.0100,0.0100,0.0100,0.0100
Fum_1234	90	0.7787,0.1530,0.0596,0.0062,0.0025	0.0100,0.0100,0.0100,0.0100,0.0100
Fum_1234	130	0.7497,0.1465,0.0767,0.0215,0.0056	0.0100,0.0100,0.0100,0.0100,0.0100
Fum_1234	250	0.7574,0.0999,0.0933,0.0429,0.0065	0.0100,0.0100,0.0100,0.0100,0.0100
Fum_1234	480	0.6988,0.1015,0.0970,0.0809,0.0218	0.0100,0.0100,0.0100,0.0100,0.0100
Fum_1234	610	0.6789,0.1017,0.1207,0.0903,0.0085	0.0100,0.0100,0.0100,0.0100,0.0100
Mal_234	20	0.8960,0.0988,0.0053,0.0000	0.0100,0.0100,0.0100,0.0100
Mal_234	40	0.8284,0.1716,0.0000,0.0000	0.0100,0.0100,0.0100,0.0100
Mal_234	60	0.7411,0.2039,0.0550,0.0000	0.0100,0.0100,0.0100,0.0100
Mal_234	90	0.6523,0.2574,0.0760,0.0144	0.0100,0.0100,0.0100,0.0100
Mal_234	130	0.5996,0.2749,0.0942,0.0312	0.0100,0.0100,0.0100,0.0100
Mal_234	250	0.3873,0.3346,0.2113,0.0667	0.0100,0.0100,0.0100,0.0100
Mal_234	480	0.3389,0.3036,0.2856,0.0719	0.0100,0.0100,0.0100,0.0100
Mal_234	610	0.3300,0.2957,0.2920,0.0823	0.0100,0.0100,0.0100,0.0100
G3P_23	20	0.9398,0.0489,0.0113	0.0100,0.0100,0.0100
G3P_23	40	0.8525,0.1271,0.0204	0.0100,0.0100,0.0100
G3P_23	60	0.7675,0.1862,0.0464	0.0100,0.0100,0.0100
G3P_23	90	0.6694,0.2526,0.0780	0.0100,0.0100,0.0100
G3P_23	130	0.5814,0.3103,0.1083	0.0100,0.0100,0.0100
G3P_23	250	0.4119,0.3952,0.1929	0.0100,0.0100,0.0100
G3P_23	480	0.3642,0.4256,0.2102	0.0100,0.0100,0.0100
G3P_23	610	0.3545,0.4274,0.2181	0.0100,0.0100,0.0100
Cit_12345	20	0.8875,0.0906,0.0201,0.0000,0.0014,0.0005	0.0100,0.0100,0.0100,0.0100,0.0100,0.0100
Cit_12345	40	0.8593,0.1300,0.0060,0.0008,0.0039,0.0000	0.0100,0.0100,0.0100,0.0100,0.0100,0.0100
Cit_12345	60	0.8370,0.1229,0.0247,0.0143,0.0001,0.0010	0.0100,0.0100,0.0100,0.0100,0.0100,0.0100
Cit_12345	90	0.7984,0.1322,0.0469,0.0225,0.0000,0.0000	0.0100,0.0100,0.0100,0.0100,0.0100,0.0100
Cit_12345	130	0.7766,0.1359,0.0555,0.0215,0.0104,0.0000	0.0100,0.0100,0.0100,0.0100,0.0100,0.0100
Cit_12345	480	0.7094,0.0863,0.0867,0.0752,0.0348,0.0077	0.0100,0.0100,0.0100,0.0100,0.0100,0.0100
Cit_12345	610	0.7568,0.0792,0.0697,0.0579,0.0324,0.0041	0.0100,0.0100,0.0100,0.0100,0.0100,0.0100

Cit_123456	20	0.8696,0.0973,0.0255,0.0020,0.0051,0.0007,0.0000	0.0110,0.0110,0.0110,0.0110,0.0110,0.0110,0.0110
Cit_123456	40	0.8287,0.1170,0.0456,0.0046,0.0031,0.0000,0.0011	0.0110,0.0110,0.0110,0.0110,0.0110,0.0110,0.0110
Cit_123456	60	0.8077,0.1138,0.0692,0.0037,0.0048,0.0010,0.0000	0.0110,0.0110,0.0110,0.0110,0.0110,0.0110,0.0110
Cit_123456	90	0.7816,0.1219,0.0540,0.0326,0.0063,0.0009,0.0028	0.0110,0.0110,0.0110,0.0110,0.0110,0.0110,0.0110
Cit_123456	130	0.7338,0.1177,0.0757,0.0530,0.0130,0.0052,0.0018	0.0110,0.0110,0.0110,0.0110,0.0110,0.0110,0.0110
Cit_123456	480	0.6931,0.0784,0.0722,0.0801,0.0518,0.0213,0.0032	0.0110,0.0110,0.0110,0.0110,0.0110,0.0110,0.0110
Cit_123456	610	0.7240,0.0949,0.0564,0.0582,0.0444,0.0198,0.0024	0.0110,0.0110,0.0110,0.0110,0.0110,0.0110,0.0110
Ga_12	20	0.7827,0.2066,0.0107	0.0100,0.0100,0.0100
Ga_12	40	0.6820,0.2939,0.0241	0.0100,0.0100,0.0100
Ga_12	60	0.6058,0.3262,0.0680	0.0100,0.0100,0.0100
Ga_12	90	0.5875,0.3344,0.0781	0.0100,0.0100,0.0100
Ga_12	130	0.5080,0.3707,0.1213	0.0100,0.0100,0.0100
Ga_12	250	0.4175,0.4121,0.1704	0.0100,0.0100,0.0100
Ga_12	480	0.4448,0.3732,0.1820	0.0100,0.0100,0.0100
Ga_12	610	0.4101,0.4066,0.1833	0.0100,0.0100,0.0100
Ga_123	20	0.7037,0.2501,0.0397,0.0066	0.0130,0.0130,0.0130,0.0130
Ga_123	40	0.6440,0.3108,0.0221,0.0232	0.0130,0.0130,0.0130,0.0130
Ga_123	60	0.5606,0.3501,0.0893,0.0000	0.0130,0.0130,0.0130,0.0130
Ga_123	90	0.4979,0.3622,0.1181,0.0219	0.0130,0.0130,0.0130,0.0130
Ga_123	130	0.3952,0.3658,0.2007,0.0384	0.0130,0.0130,0.0130,0.0130
Ga_123	250	0.3317,0.3341,0.2522,0.0821	0.0130,0.0130,0.0130,0.0130
Ga_123	480	0.3652,0.2964,0.2428,0.0957	0.0130,0.0130,0.0130,0.0130
Ga_123	610	0.2982,0.3347,0.2784,0.0887	0.0130,0.0130,0.0130,0.0130

Table S10. Experimental exchange fluxes for flux estimation of the *Synechocystis* model.

Reaction ID	Mean (μmol $gCDW^{-1}$ s^{-1})	SD
co2in	13.32	0.01

Table S11. Comparison of the dilution parameters (G-values) estimated by FreeFlux and Metran for the *E. coli* model.

Dilution	FreeFlux	Metran
Ala	98%	98%
Gly	59%	57%
Val	98%	98%
Leu	97%	97%
Ile	98%	98%
Ser	98%	98%
Phe	98%	98%
Asp	97%	98%
Glu	97%	97%
Tyr	100%	100%
CO_2 [a]	26.1	19.3

[a] CO_2 dilution denotes a co2_d flux normalized to a glucose uptake rate of 100.

Table S12. Comparison of the dilution parameters (G-values) estimated by FreeFlux and INCA for the *Synechocystis* model.

Dilution	FreeFlux	INCA
DHAP	92%	93%
F6P	92%	90%
GAP	91%	90%
R5P	93%	92%

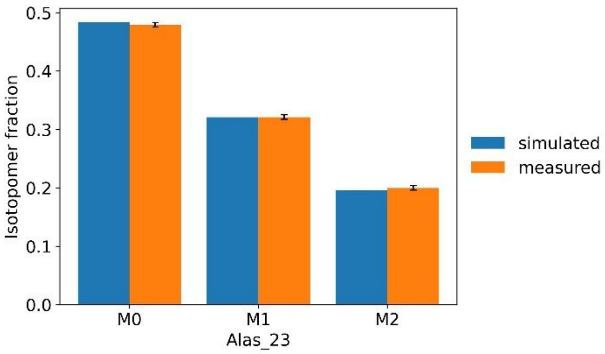
Isotopomer fraction
M0
M1
M2
Alas_23
simulated
measured

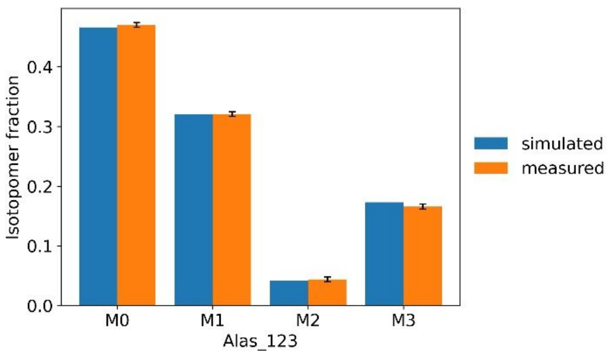
Isotopomer fraction
M0
M1
M2
M3
Alas_123
simulated
measured

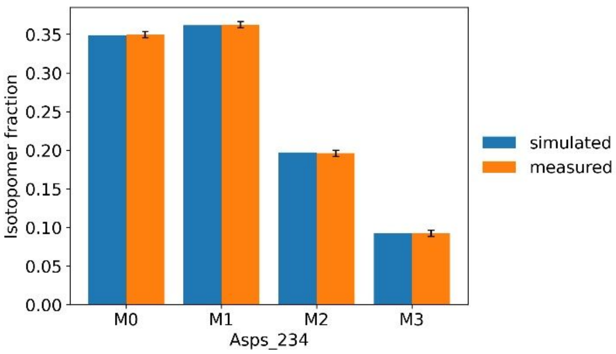
Isotopomer fraction
M0
M1
M2
M3
Asps_234
simulated
measured

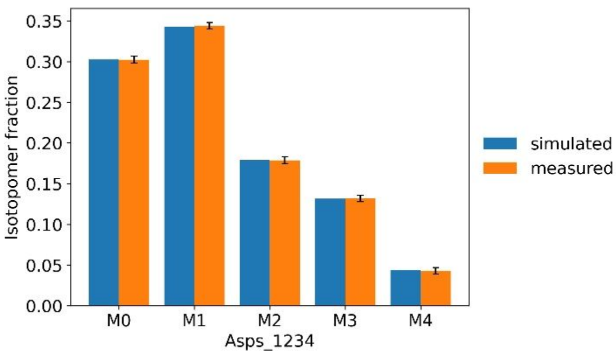
Isotopomer fraction
M0
M1
M2
M3
M4
Asps_1234
simulated
measured

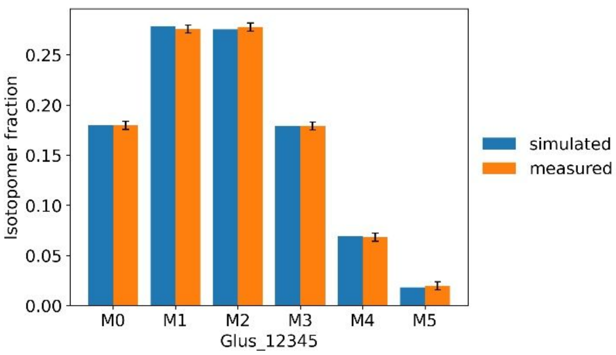
Isotopomer fraction
M0
M1
M2
M3
M4
M5
Glus_12345
simulated
measured

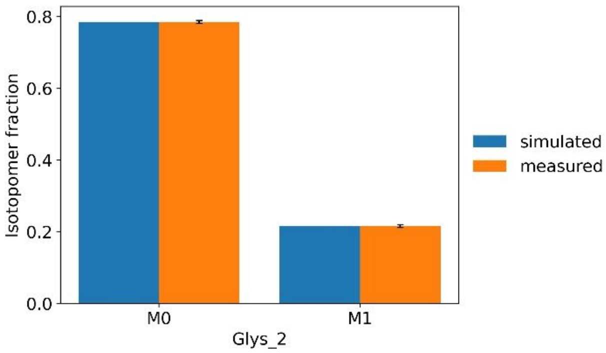
Isotopomer fraction
M0
M1
Glys_2
simulated
measured

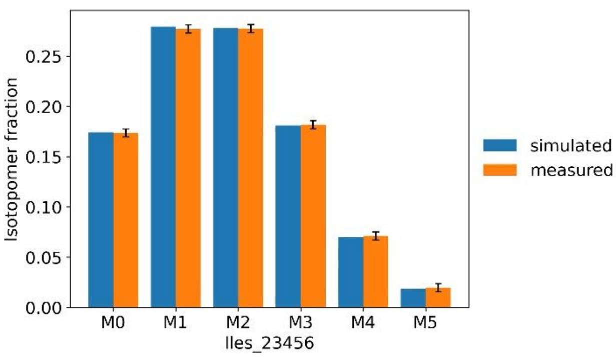
Isotopomer fraction
M0
M1
M2
M3
M4
M5
Iles_23456
simulated
measured

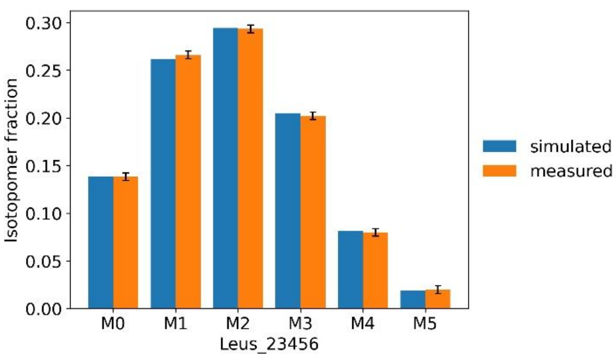
Isotopomer fraction
M0
M1
M2
M3
M4
M5
Leus_23456
simulated
measured

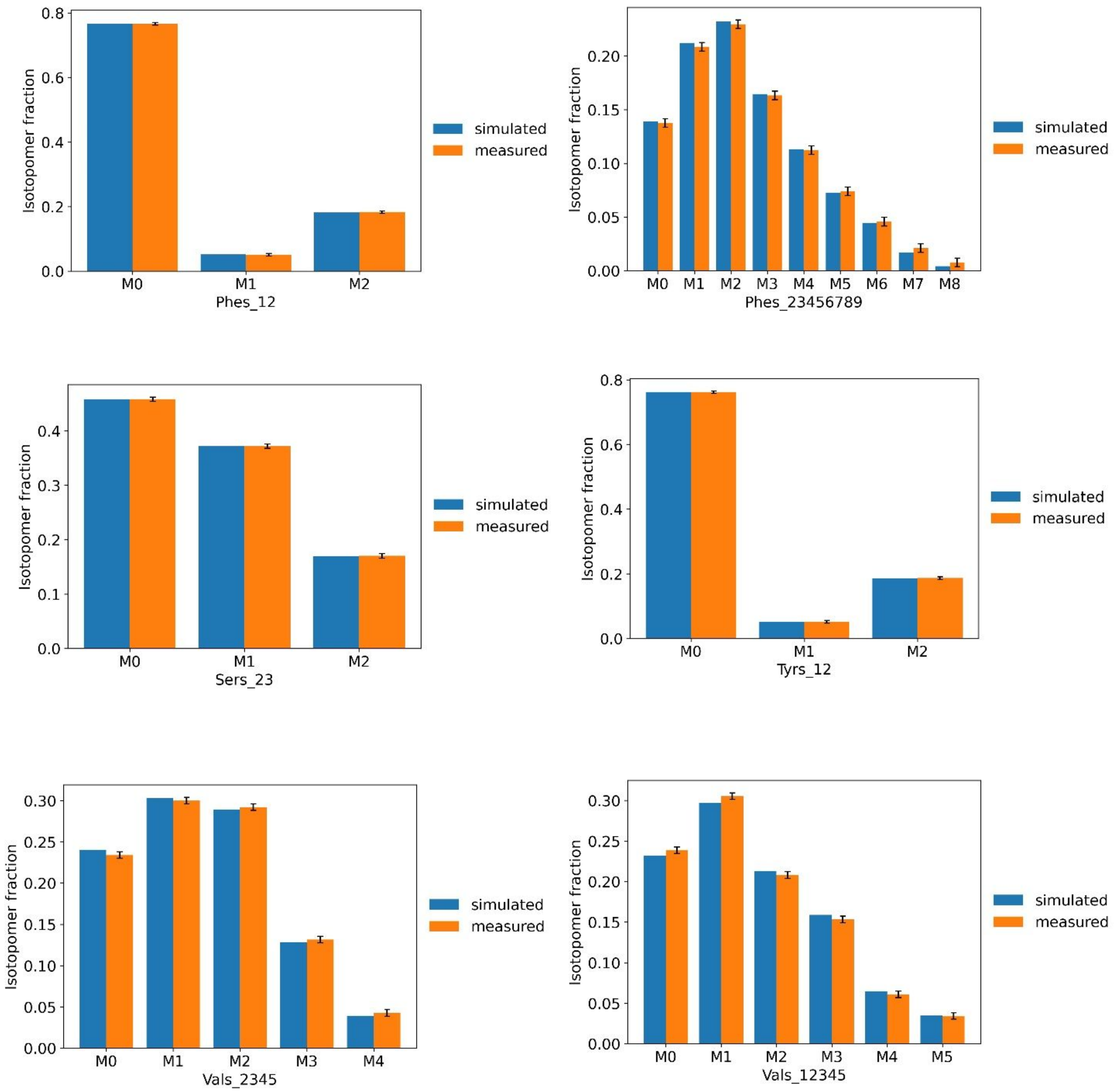

Figure S1. comparison of experimental and simulated MDVs at convergence in flux estimation of the *E. coli* model. The suffix “s” after amino acid denotes corresponding sampling pool, as dilution effect of unlabeled pools of these metabolites were considered (Table S1).

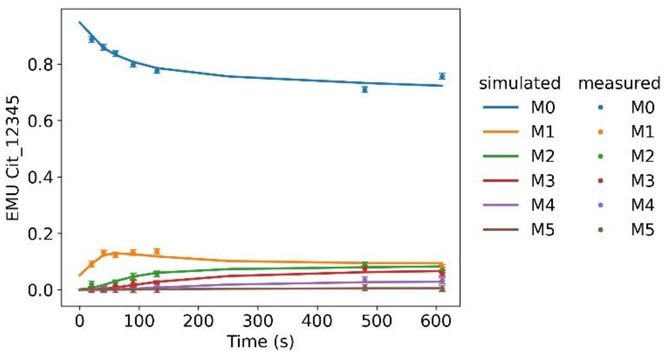
EMU Cit_12345
Time (s)
simulated
measured
M0
M1
M2
M3
M4
M5

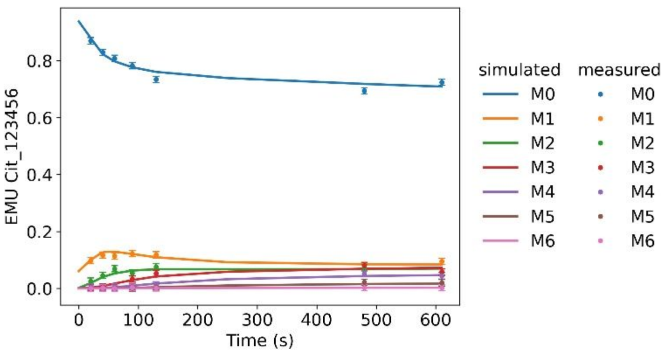
EMU Cit_123456
Time (s)
simulated
measured
M0
M1
M2
M3
M4
M5
M6

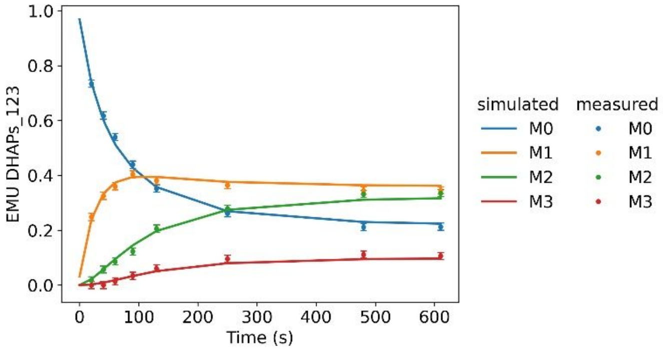
EMU DHAPs_123
Time (s)
simulated
measured
M0
M1
M2
M3

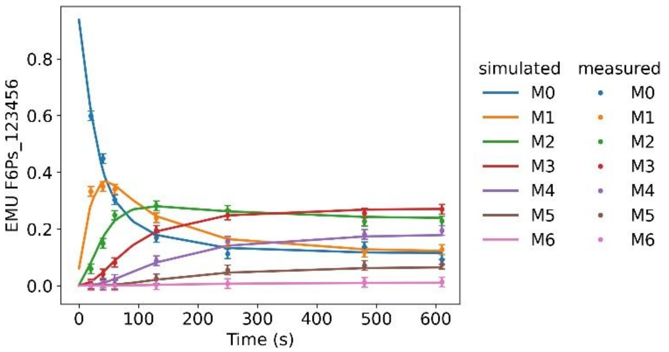
EMU F6Ps_123456
Time (s)
simulated
measured
M0
M1
M2
M3
M4
M5
M6

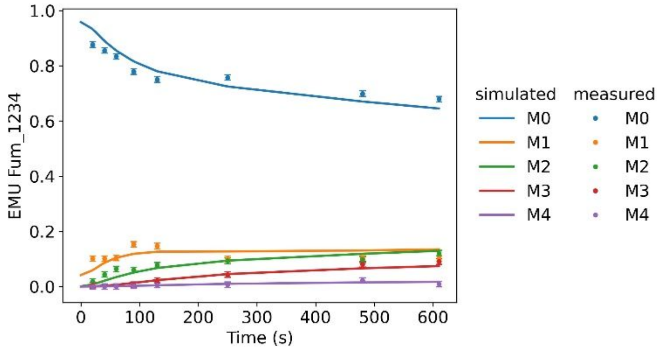
EMU Fum_1234
Time (s)
simulated
measured
M0
M1
M2
M3
M4

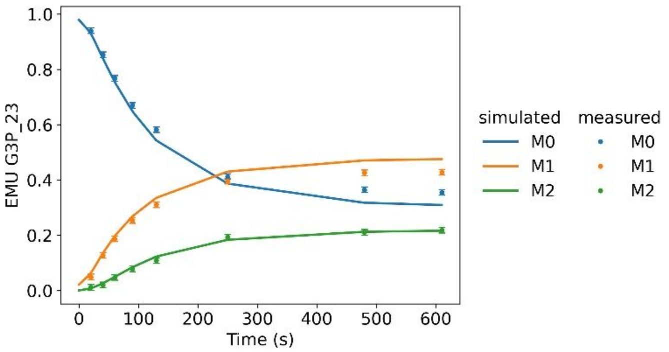
EMU G3P_23
Time (s)
simulated
measured
M0
M1
M2

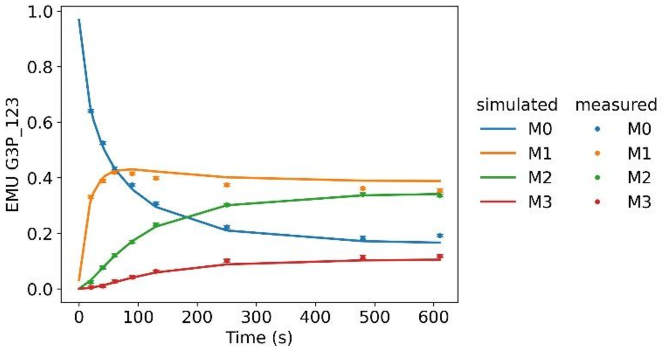
EMU G3P_123
Time (s)
simulated
measured
M0
M1
M2
M3

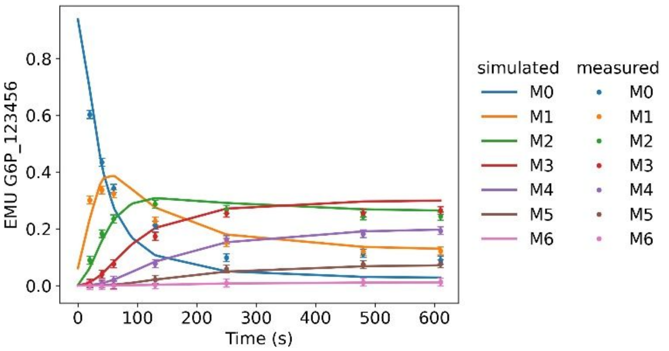
EMU G6P_123456
Time (s)
simulated
measured
M0
M1
M2
M3
M4
M5
M6

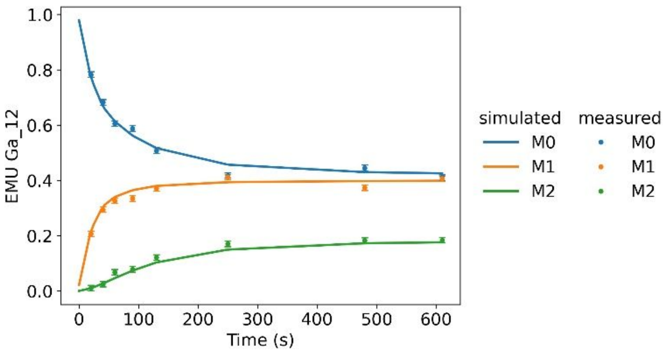
EMU Ga_12
Time (s)
simulated
measured
M0
M1
M2

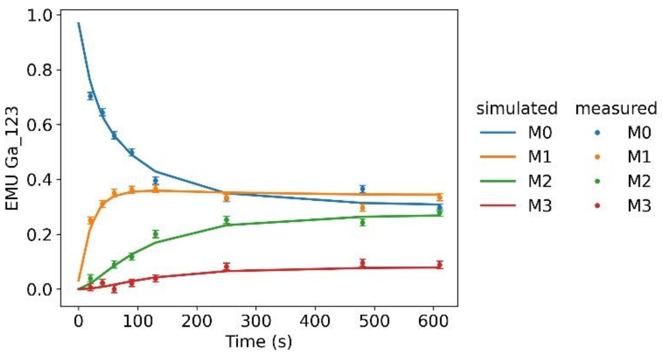
EMU Ga_123
Time (s)
simulated
measured
M0
M1
M2
M3

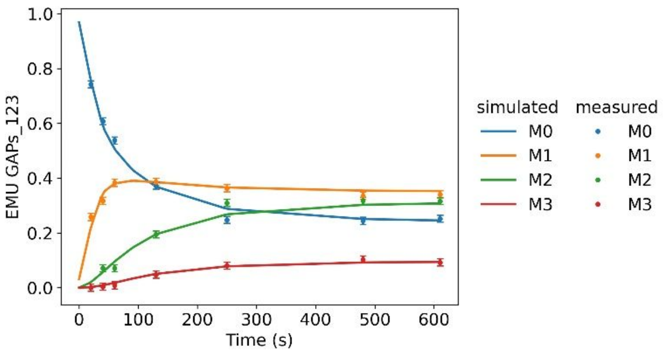
EMU GAPs_123
Time (s)
simulated
measured
M0
M1
M2
M3

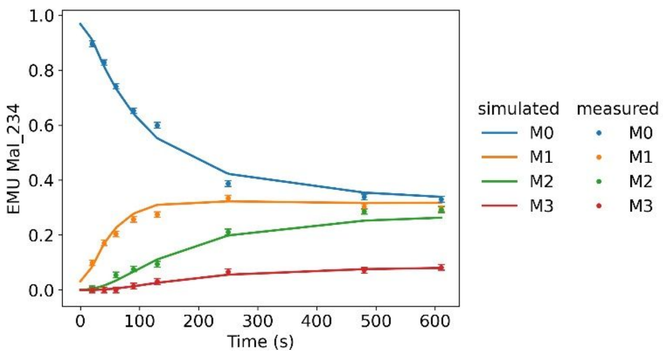
EMU Mal_234
Time (s)
simulated
measured
M0
M1
M2
M3

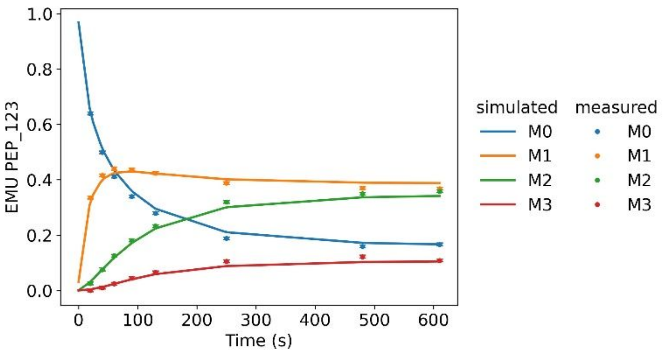
EMU PEP_123
Time (s)
simulated
measured
M0
M1
M2
M3

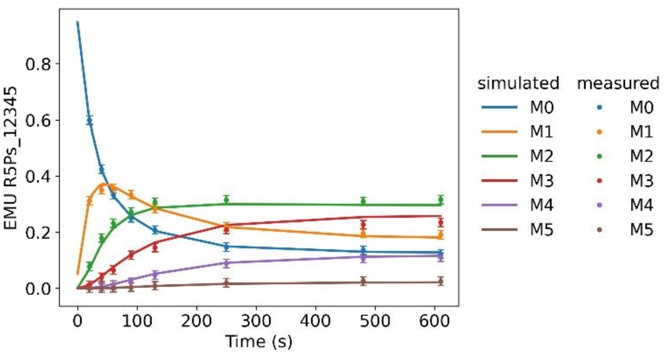
EMU R5Ps_12345
Time (s)
simulated
measured
M0
M1
M2
M3
M4
M5

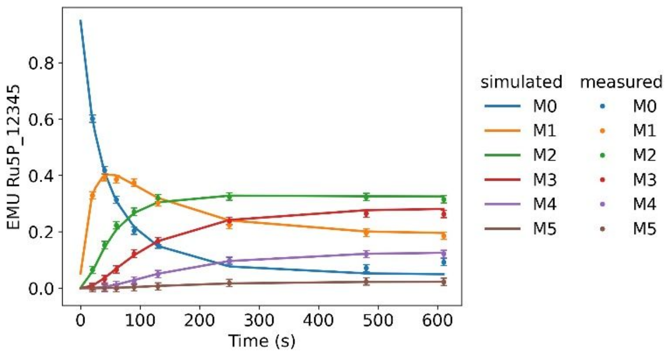
EMU Ru5P_12345
Time (s)
simulated
measured
M0
M1
M2
M3
M4
M5

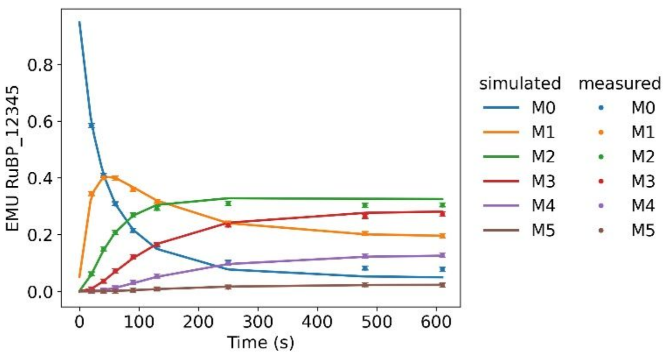
EMU RuBP_12345
Time (s)
simulated
measured
M0
M1
M2
M3
M4
M5

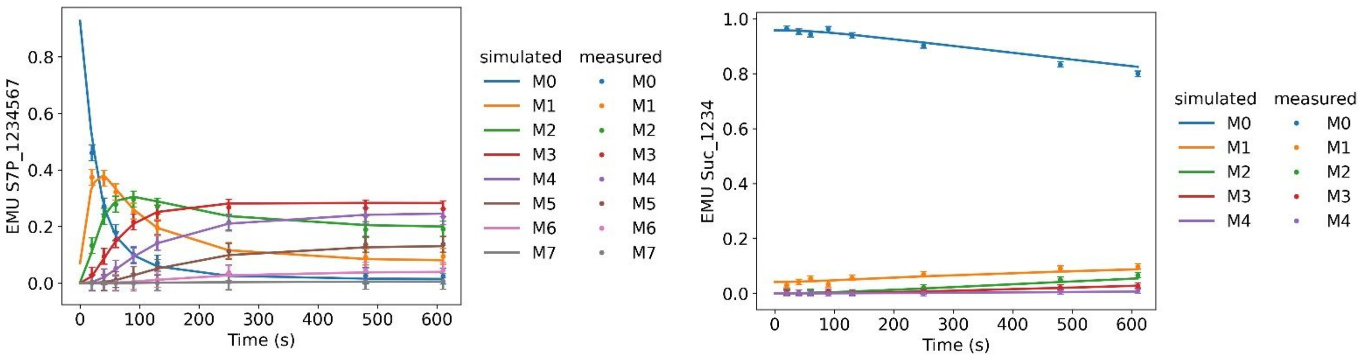

Figure S2. comparison of time-course experimental and simulated MDVs at convergence in flux estimation of the *Synechocystis* model. The suffix "s" after amino acid denotes corresponding sampling pool, as dilution effect of unlabeled pools of these metabolites were considered (Table S6).
